# Implementation of the Comprehensive Unit-Based Safety Program to Improve Infection Prevention and Control Practices in Four Neonatal Intensive Care Units in Pune, India

**DOI:** 10.3389/fped.2021.794637

**Published:** 2022-01-06

**Authors:** Julia Johnson, Asad Latif, Bharat Randive, Abhay Kadam, Uday Rajput, Aarti Kinikar, Nandini Malshe, Sanjay Lalwani, Tushar B. Parikh, Umesh Vaidya, Sudhir Malwade, Sharad Agarkhedkar, Melanie S. Curless, Susan E. Coffin, Rachel M. Smith, Matthew Westercamp, Elizabeth Colantuoni, Matthew L. Robinson, Vidya Mave, Amita Gupta, Yukari C. Manabe, Aaron M. Milstone

**Affiliations:** ^1^Division of Neonatology, Department of Pediatrics, Johns Hopkins University School of Medicine, Baltimore, MD, United States; ^2^Department of International Health, Johns Hopkins Bloomberg School of Public Health, Baltimore, MD, United States; ^3^Department of Anesthesia and Critical Care Medicine, Johns Hopkins University School of Medicine, Baltimore, MD, United States; ^4^Armstrong Institute for Patient Safety and Quality, Johns Hopkins Medicine, Baltimore, MD, United States; ^5^Byramjee-Jeejeebhoy Government Medical College-Johns Hopkins University Clinical Research Site, Pune, India; ^6^Department of Pediatrics, Byramjee-Jeejeebhoy Government Medical College, Pune, India; ^7^Department of Neonatology, Bharati Vidyapeeth Deemed to Be University Medical College, Pune, India; ^8^Department of Pediatrics, Bharati Vidyapeeth Deemed to Be University Medical College, Pune, India; ^9^Department of Pediatrics, King Edward Memorial Hospital, Pune, India; ^10^Department of Pediatrics, Dr. D. Y. Patil Medical College, Pune, India; ^11^Department of Hospital Epidemiology and Infection Control, Johns Hopkins Hospital, Baltimore, MD, United States; ^12^Division of Infectious Diseases, Department of Pediatrics, University of Pennsylvania, Philadelphia, PA, United States; ^13^Centers for Disease Control and Prevention, Atlanta, GA, United States; ^14^Department of Biostatistics, Bloomberg School of Public Health, Johns Hopkins University, Baltimore, MD, United States; ^15^Division of Infectious Diseases, Department of Medicine, Johns Hopkins University School of Medicine, Baltimore, MD, United States; ^16^Division of Pediatric Infectious Diseases, Department of Pediatrics, Johns Hopkins University School of Medicine, Baltimore, MD, United States

**Keywords:** neonate, healthcare-associated infection, patient safety, hand hygiene, aseptic technique, patient safety culture, multimodal strategy, bloodstream infection

## Abstract

**Objective:** To implement the Comprehensive Unit-based Safety Program (CUSP) in four neonatal intensive care units (NICUs) in Pune, India, to improve infection prevention and control (IPC) practices.

**Design:** In this quasi-experimental study, we implemented CUSP in four NICUs in Pune, India, to improve IPC practices in three focus areas: hand hygiene, aseptic technique for invasive procedures, and medication and intravenous fluid preparation and administration. Sites received training in CUSP methodology, formed multidisciplinary teams, and selected interventions for each focus area. Process measures included fidelity to CUSP, hand hygiene compliance, and central line insertion checklist completion. Outcome measures included the rate of healthcare-associated bloodstream infection (HA-BSI), all-cause mortality, patient safety culture, and workload.

**Results:** A total of 144 healthcare workers and administrators completed CUSP training. All sites conducted at least 75% of monthly meetings. Hand hygiene compliance odds increased 6% per month [odds ratio (OR) 1.06 (95% CI 1.03–1.10)]. Providers completed insertion checklists for 68% of neonates with a central line; 83% of checklists were fully completed. All-cause mortality and HA-BSI rate did not change significantly after CUSP implementation. Patient safety culture domains with greatest improvement were management support for patient safety (+7.6%), teamwork within units (+5.3%), and organizational learning—continuous improvement (+4.7%). Overall workload increased from a mean score of 46.28 ± 16.97 at baseline to 65.07 ± 19.05 at follow-up (*p* < 0.0001).

**Conclusion:** CUSP implementation increased hand hygiene compliance, successful implementation of a central line insertion checklist, and improvements in safety culture in four Indian NICUs. This multimodal strategy is a promising framework for low- and middle-income country healthcare facilities to reduce HAI risk in neonates.

## Introduction

As facility-based births increase worldwide, low- and middle-income countries (LMIC) increasingly provide care for premature and sick neonates in neonatal intensive care units (NICUs) and special care nurseries (1). Hospitalized neonates are uniquely vulnerable to healthcare-associated infections (HAI) (2, 3). Poor infection prevention and control (IPC) practices augment this risk in many LMIC healthcare facilities (4). The burden of HAI in hospitalized neonates in LMICs exceeds that of facilities in high-income settings (5). The predominance of multi-drug resistant Gram-negative HAIs, which have limited treatment options and are associated with high morbidity and mortality in neonates, underscores the urgency of prevention interventions (6–8).

The World Health Organization (WHO) recommends implementation of multimodal improvement strategies for IPC with the following five elements: (1) system change, (2) training and education, (3) monitoring and feedback, (4) reminders and communication, and (5) culture change (9, 10). The Comprehensive Unit-based Safety Program (CUSP) is a multimodal improvement strategy that has been successfully implemented to improve IPC practices. CUSP has been used to reduce risk of central line-associated bloodstream infections (CLABSIs) and other HAIs in a variety of healthcare settings and populations (11–17). CUSP fosters the creation of a unit-based multidisciplinary team and empowers staff to assume responsibility for change, improving local patient safety culture and compliance with best practices to reduce HAIs and other threats to patient safety (18). While CUSP has been successfully applied internationally in high-income settings, including in Saudi Arabia and the United Arab Emirates, there is limited experience in NICU and LMIC settings (16, 17). Our objective was to assess the performance of CUSP in NICUs in an LMIC setting to guide IPC improvement strategies, reduce HAI risk in hospitalized neonates, and improve patient safety culture.

## Methods

In this quasi-experimental study, we implemented CUSP to improve adherence to evidence-based IPC practices in four tertiary care NICUs in Pune, India. Consent was obtained from all healthcare workers (HCWs) who completed surveys. This study was approved by the Johns Hopkins Medicine Institutional Review Board, the ethics committees of all participant sites, and the Indian Health Ministry's Screening Committee. This manuscript uses the SQUIRE 2.0 standards for reporting (19).

### Study Sites

All participant sites are tertiary care facilities with high-volume NICUs located in Pune, India, though referral patterns and patient demographics differ. Byramjee Jeejeebhoy Government Medical College (BJGMC) is a government medical college affiliated with Sassoon Hospital, which has a 60-bed NICU. King Edward Memorial (KEM) Hospital is a non-governmental facility run by a charitable trust and has a 46-bed NICU. Dr. D. Y. Patil Medical College is a private medical college and has a 26-bed NICU. Bharati Vidyapeeth Deemed University Medical College is a private medical college and has a 60-bed NICU. All hospitals are delivery centers and admit both inborn and outborn neonates.

### CUSP Implementation

**Figure 1:**
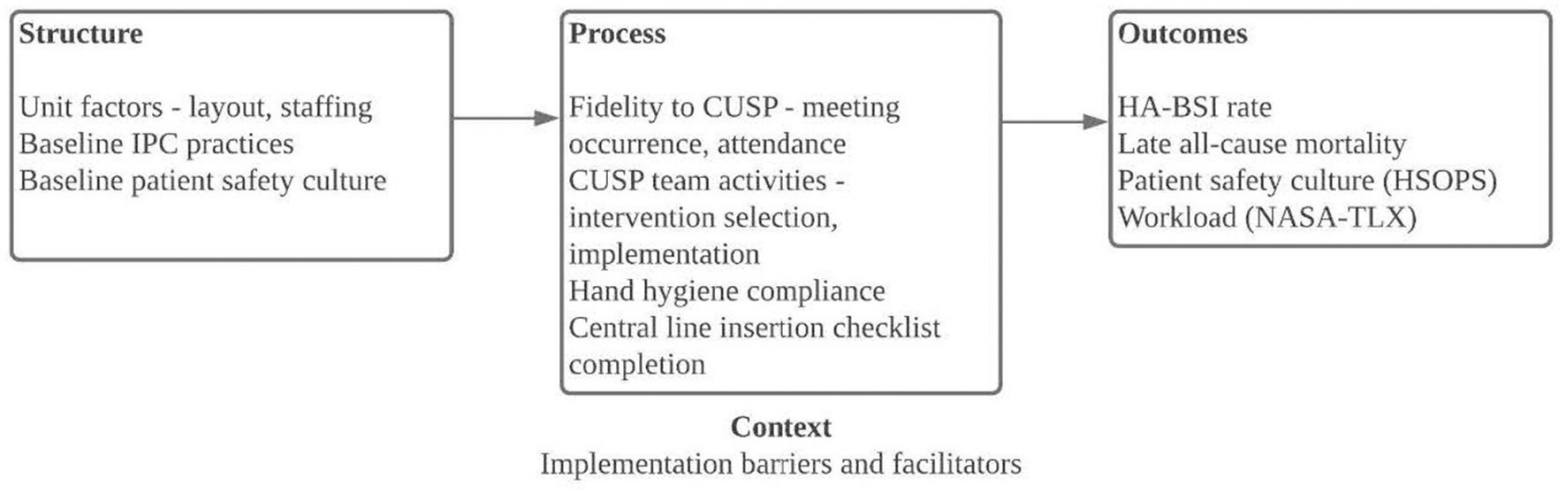
Conceptual framework for CUSP implementation at four tertiary care neonatal intensive care units in Pune, India. CUSP, Comprehensive Unit-based Safety Program; HA-BSI, healthcare-associated bloodstream infections; HSOPS, Hospital Survey on Patient Safety Culture; IPC, infection prevention and control; NASA-TLX, National Aeronautics and Space Administration Task Load Index.

CUSP is a validated strategy to empower staff to improve unit-level patient safety and consists of the following steps: (1) educate staff on the science of safety, (2) engage staff in identifying defects, (3) partner with a senior executive, (4) identify and learn from defects, and (5) implement teamwork tools (20). CUSP methodology has previously been described in the literature and the CUSP toolkit is publicly available on the Agency for Healthcare Research and Quality's (AHRQ) website at www.ahrq.gov (11, 20, 21). In this study, CUSP was used to improve HCW adherence to evidence-based IPC practices and reduce HAI risk in the NICU (see conceptual framework, Figure 1). Site staff received training in CUSP methodology and formed multidisciplinary teams led by a CUSP nurse champion and physician champion. Monthly CUSP meetings were attended by CUSP facilitators. Teams were additionally supported by monthly coaching calls.

### Baseline IPC Assessments

Baseline IPC assessments of practices relevant to neonatal care at the facility level and within the Labor & Delivery ward and NICU were conducted in February 2017 and January 2018 using the Infection Control Assessment Tool (ICAT), 2nd version (2009) (22, 23). We supplemented the ICAT with questions specific to neonatal care, such as the storage and preparation of breast milk and formula feeds, umbilical catheter insertion and maintenance, and isolette and radiant warmer cleaning and disinfection. After review of these assessments and discussions with key local stakeholders, we identified three focus areas for CUSP: hand hygiene, aseptic technique for invasive procedures, and medication and intravenous (IV) fluid preparation and administration. For the third focus area, medication and IV fluid preparation and administration, sites were additionally provided an injection safety assessment tool that guided site self-assessment of existing practices (see Supplementary Material).

### Selection and Implementation of Interventions

After CUSP teams were formed, unit-level staff selected interventions for the three focus areas, hand hygiene, aseptic technique for invasive procedures, and medication and IV fluid preparation and administration. For each pre-selected focus area, sites completed a two-question Staff Safety Assessment (SSA), adapted from the CUSP toolkit to each focus area in order to elicit staff perceptions of threats to patient safety within each focus area (Supplementary Table 1). After analyzing SSA responses, each CUSP team identified and implemented targeted interventions for each focus area. All sites initiated hand hygiene monitoring and implemented a central line insertion checklist (see Supplementary Material). Other interventions were selected and implemented at sites' discretion (Table 1). For focus area 2, creation of a central line maintenance audit tool was requested; completion of this tool was encouraged but not monitored (see Supplementary Material). For focus area 3, the injection safety assessment tool was used to support site assessments of existing practices and selected of interventions, which included use of multi-dose medication vials with a lower concentration, thereby reducing number of doses per vial, use of dedicated staff for preparation of all medications and IV fluids, and moving medication and IV fluid preparation to a separate space outside of the immediate patient care area (Figure 1). Notably, most medications administered in site NICUs are admixed in the patient care areas, rather than in the hospital's central pharmacy.

**Table 1:** CUSP interventions, general and by focus area, categorized by the five elements of the WHO multimodal IPC improvement strategy.

	**System change**	**Training and education**	**Monitoring and feedback**	**Reminders and communication**	**Culture of safety**
General	Creation of multidisciplinary CUSP team^*^ Senior executive linked with unit^*^ Infection control member of CUSP team^*^ CUSP nurse to support interventions^*^ Dedicated unit staff to decrease turnover	CUSP methodology training for administrators and unit-level staff^*^ CUSP orientation for new unit staff Transition training for CUSP sustainability^*^ HA-BSI root cause analysis training^*^	HA-BSI rate monitoring^*^ Sharing of HA-BSI data at monthly CUSP meetings^*^ Monitoring of CUSP meeting frequency, attendance, and participation^*^	Use of WhatsApp group to facilitate CUSP champion communication with study team^*^ Use of WhatsApp groups to announce CUSP meetings, circulate agenda, and distribute information^*^ Monthly CUSP coaching calls^*^	Creation of CUSP mission statement focused on patient safety^*^ CUSP team logo design Display of mission and logo in unit Engagement of staff in identifying threats to safety via SSA^*^
Hand hygiene	Involvement of mothers and families in HH Change in HH product to reduce allergic dermatitis Assessment of supply chain issues Senior executive involvement in addressing supply chain issues Creation of additional ABHR storage space to avoid stock outage Emergency cart stocked with HH supplies	Group HH demonstration sessions Video-based HH education Targeted training for HH moments with poor compliance^*^ Training for new interns by senior residents Training of mothers on HH technique Involvement of mothers in education of other mothers	Internal HH compliance monitoring using SpeedyAudit^TM^ application^*^ Sharing of HH data at monthly CUSP meetings^*^ Feedback of HH data to unit staff^*^ Individualized feedback for those with poor compliance CCTV use for targeted HH monitoring and feedback Appreciation of unit staff following best HH practices	Posters describing five moments of HH and importance of HH Bedside HH reminders Televised reminders in staff areas	HH SSA completion^*^ HH focused mission statement^*^ Emphasis on importance of HH by all unit staff^*^ Coordination with other departments to motivate visiting staff to perform HH Participation in World Hand Hygiene Day^*^
Aseptic technique	Implementation of central line insertion checklist^*^ Implementation of central line maintenance checklist^*^	Use of slide presentation and videos to train staff on aseptic technique^*^ NABH care bundle training materials Training for new interns by senior residents	Monthly audits of central line insertion checklist completion^*^ Sharing of checklist completion data at monthly CUSP meetings^*^	WhatsApp group reminders for CL insertion and maintenance checklist completion Use of WhatsApp groups to circulate training materials, posters, and NABH care bundle information	Aseptic technique SSA completion^*^ Nurses/staff empowered to stop aseptic procedure if appropriate steps not followed^*^
Medication and IV fluids	Dedicated staff assigned to prepare all injections Dedicated injection preparation space outside of immediate patient care area Change in use of multi-dose vials with lower medication concentration when available Additional refrigerated storage for medications Change in standard injection times to reduce workload	Use of slide presentation to train staff on injection safety^*^ Training for new interns by senior residents	Observation of hub cleaning practices Injection safety audit^*^	Posters detailing steps of medication preparation Bedside reminder flags for scrubbing the hub	Medication and intravenous fluid preparation and administration SSA completion^*^

*Interventions were selected and implemented by CUSP teams and site-specific*.

* *Interventions implemented by all sites. ABHR, alcohol-based hand rub; CL, central line; CUSP, Comprehensive Unit-based Safety Program; HH, hand hygiene; IPC, infection prevention and control; IV, intravenous; NABH, National Accreditation Board of Hospitals and Healthcare Providers (India); SSA, Staff Safety Assessment; WHO, World Health Organization*.

During monthly CUSP coaching calls, study team CUSP coaches and facilitators introduced adapted CUSP tools to support the implementation of interventions, such as the SSA and Patient Safety Rounds (see Supplementary Material) (21).

### Outcomes

Process measures included CUSP training participation, monthly meeting occurrence and attendance, hand hygiene compliance, and central line insertion checklist completion. Outcome measures included the rate of healthcare-associated bloodstream infections (HA-BSI), all-cause mortality, patient safety culture, and workload.

### Process Measures

#### CUSP Participation

We recorded attendance of all participants in CUSP training by name, role, and site. Each site recorded attendance of all participants by name and role at each monthly CUSP meeting.

#### Hand Hygiene Compliance

Hand hygiene compliance was measured by direct observation using trained external observers via the SpeedyAudit^TM^ application (HandyMetrics Corporation, Toronto, Canada). Hand hygiene compliance was recorded by HCW role and the five moments of hand hygiene: (1) before touching a patient; (2) before clean/aseptic procedures; (3) after body fluid exposure risk; (4) after touching a patient; and (5) after touching patient surroundings (24). Hand hygiene data by HCW role and moment of hand hygiene were reported monthly and shared with site CUSP teams and unit staff throughout the study period.

#### Central Line Insertion Checklist Completion

The central line insertion checklist was adapted from the Johns Hopkins Hospital Pediatric Central Arterial and Venous Catheter Insertion Checklist with input from local stakeholders (see tools included in Supplementary Material). Sites implemented the central line insertion checklist in January-February 2019; none of the sites had a similar checklist in place prior to the study. Checklist completion was monitored by monthly audit completed by external assessors, with an assessment of central line checklist presence and completion in the medical record of neonates with a central line in place, and reported monthly at CUSP meetings.

### Outcome Measures

#### Healthcare-Associated Bloodstream Infections and All-Cause Mortality

HA-BSI was defined as culture-confirmed BSI on hospital day 3 or greater. The monthly rate of HA-BSI was expressed as cases per 1,000 patient-days. Blood cultures were obtained at the discretion of the clinical teams and processed at site microbiology laboratories. For the primary outcome of HA-BSI, organisms deemed as likely contaminants (per categorization as a common commensal per the Centers for Disease Control and Prevention National Healthcare Safety Network) were excluded, with the exception of coagulase negative Staphylococcus (CONS), one of the most common HA-BSI pathogens in hospitalized neonates (2, 25). All-cause mortality was defined as the number of deaths per 100 admissions among neonates admitted for at least 3 days. HA-BSI and mortality data was collected as part of a concurrent prospective cohort study enrolling all neonates admitted to the NICU in which three sites participated (26). The fourth site reported unit-level HA-BSI and mortality data for outcome ascertainment.

#### Patient Safety Culture

Patient safety culture was assessed at baseline and follow-up using the Agency for Healthcare Research and Quality (AHRQ) Surveys on Patient Safety Culture (SOPS^TM^) Hospital Survey (HSOPS) version 1.0 (27). The HSOPS consists of 42 items organized into 12 composite dimensions that assess elements of patient safety culture using a Likert response scale as well as nine items assessing respondent characteristics (Supplementary Table 2). The survey was administered in English, Marathi, or Hindi based on respondent preference. Survey responses were anonymous, and baseline and follow-up survey responses were not linked.

#### Workload

Workload was assessed at baseline and follow-up using the National Aeronautics and Space Administration Task Load Index (NASA-TLX) (28). The NASA-TLX assesses workload across six domains: mental demand, physical demand, temporal demand, effort, performance, and frustration. Each domain is assessed by a single item on a 20-point continuous scale. The NASA-TLX was administered with the HSOPS; responses were also anonymous and not linked.

### Statistical Analysis

Logistic regression models were used to describe trends in hand hygiene compliance after CUSP implementation. The models included month and allowed for comparisons across site and by HCW role. All regression models accounted for potential autoregressive correlation of rates within a site over time; standard errors for the pooled analyses were estimated using robust variance estimates.

Descriptive analyses included summarizing baseline and post-implementation HA-BSI rates and all-cause mortality overall and by site. The site-specific baseline and post-implementation HA-BSI and mortality rates were compared using two-sample Poisson tests. The pooled relative monthly HA-BSI and mortality rates comparing the post-implementation and baseline periods were estimated using Poisson regression models with the number of HA-BSI or deaths as the outcome, main effect for the post-implementation and baseline periods and offset for total exposure time (patient-days or admissions).

For HSOPS analysis, percent positive scores (PPS) by item were calculated by dichotomizing responses and reverse coding scores for negative items. Mean PPS for composite domains were calculated by averaging PPS across items included in each domain. Comparison of baseline and follow-up patient safety culture was performed by site-level analysis of the difference in mean PPS for each composite domain. Confidence intervals (CIs) for the change in the mean PPS for composite domains were generated using a bootstrap procedure. Given that it was not possible to link responses for a respondent who participated in both baseline and follow-up surveys, the bootstrap procedure was used to replicate this potential clustering by resampling respondents with replacement within site and pre- and post-intervention surveys. The reported bootstrap CIs are based on 1000 bootstrap samples and use the bias-corrected and accelerated method.

For NASA-TLX analysis, mean scores were calculated at baseline and follow-up for the six domains of workload. An overall workload score was calculated by summing the six domain scores at baseline and at follow-up, for a maximum score of 120. Baseline and post-intervention means were compared using Student's *t*-test. A *p*-value < 0.05 was considered statistically significant.

All statistical analyses were completing using Stata version 15.0 (Stata Corp., College Station, TX) and R version 3.6.1 (R Foundation for Statistical Computing, Vienna, Austria).

## Results

### Process Measures

#### CUSP Methodology Training

Across the four sites, 144 HCWs and administrators participated in CUSP methodology training in March 2018. Central training was attended mostly by administrators and senior leadership, as well as physician and nurse champions for each site. Site-based training included primarily unit-based staff as well as infection control staff. By the conclusion of training, all sites identified CUSP team members, including physician and nurse champions, a senior executive partner, infection control staff, and additional unit-based physicians and nurses.

#### CUSP Meetings and Coaching Calls

CUSP meetings took place monthly at each site over the course of the study period (Table 2). All sites conducted at least 75% of monthly meetings, with average attendance ranging from 8.8 to 14.8 participants per meeting across sites. The proportion of monthly meetings attended was highest among physician and nurse champions (87–100%). Senior executive attendance varied from 7% to 75%.

**Table 2:** CUSP team meeting frequency and attendance by site and month, June 2018-September 2019.

	**Site 1**	**Site 2**	**Site 3**	**Site 4**
Meetings took place, n (%)	16 (100)	15 (94)	12 (75)	12 (75)
Number of attendees, mean	11.7	14.8	8.8	12.1
Meetings attended by, n (%)
Physician champion	16 (100)	15 (100)	11 (92)	12 (100)
Nurse champion	16 (100)	13 (87)	11 (92)	12 (100)
Senior nurse	14 (88)	5 (33)	0	8 (67)
Infection control	7 (44)	13 (87)	9 (75)	12 (100)
Senior executive	4 (25)	1 (7)	6 (50)	9 (75)
CUSP facilitator	16 (100)	15 (100)	11 (92)	11 (92)

*Number of attendees (summary and by month) includes only meeting participants from the site, not the CUSP facilitator or any other study staff. CUSP facilitator attendance is noted separately. CUSP, Comprehensive Unit-based Safety Program*.

#### Hand Hygiene Compliance

**Figure 2:**
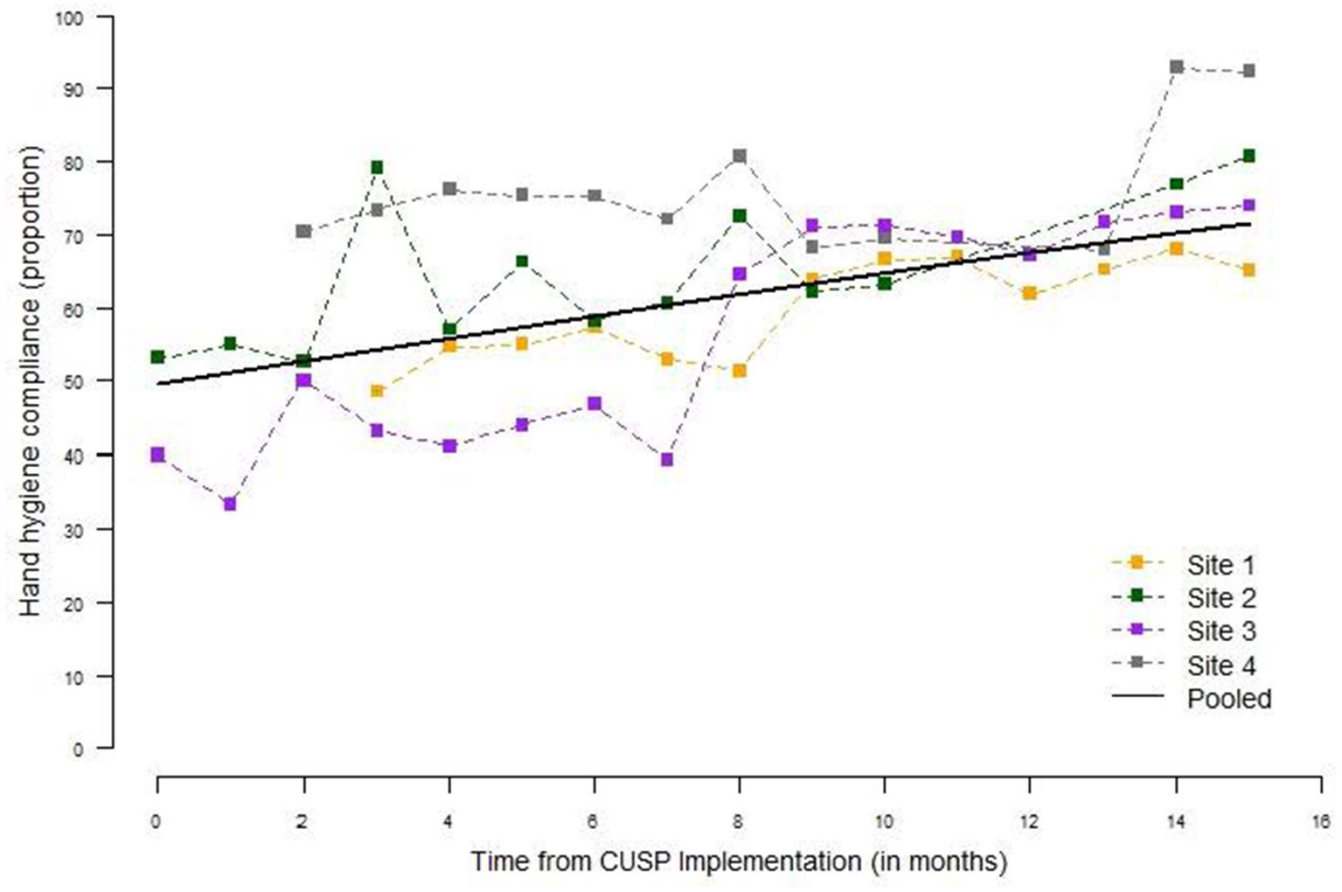
Hand hygiene compliance by site and month, June 2018–September 2019. Monthly hand hygiene compliance was expressed as the proportion of monthly observations compliant, by site for all healthcare worker roles. Hand hygiene was monitored monthly via direct observation by trained observers. There were 8,684 opportunities for hand hygiene across all four sites over the course of the study period. The pooled hand hygiene compliance is based on a logistic regression model for observation compliance as a function of month from CUSP implementation.

There were 8684 hand hygiene observations across the sites over the course of the study period with all four sites collecting hand hygiene observations within 3 months of CUSP implementation (Figure 2). From the pooled analysis of all four sites, the proportion of compliant hand hygiene observations during the month of CUSP implementation was 51% (95% confidence interval (CI) 40-62%) and increased significantly to 56% (95% CI 46–65%), 65% (95% CI 57–72%), and 73% (95% CI 65–81%) by the 3rd, 9th and 15th month, respectively, following CUSP implementation; odds of hand hygiene compliance increased 6% per month, odds ratio (OR) 1.06, 95% CI 1.03–1.10). All sites had a statistically significant increase in the estimated hand hygiene compliance from CUSP implementation to the 15th month thereafter: site 1 from 48 to 67% (OR 1.05, 95% CI 1.04–1.07), site 2 from 52 to 78% (OR 1.08, 95% CI 1.06–1.11), site 3 from 33 to 76% (OR 1.13, 95% CI 1.10–1.16), and site 4 from 61 to 87% (OR 1.10, 95% CI 1.01–1.19). The rate of change of hand hygiene compliance over time did not differ by HCW role (*p* = 0.988).

#### Central Line Insertion Checklist Completion

From January 2019 until September 2019, there were 486 neonates who had a central line in place at time of monthly checklist audit. For site 1 (*n* = 146), 68% of neonates with a central line in place had an insertion checklist present in the medical record; site 2 (*n* = 166) 100%, site 3 (*n* = 136) 48%, and site 4 (*n* = 38) 66%. Site 4's data were only representative of the last 5 months of checklist use; data were collected on checklist presence and completion, but not on the total number of neonates with central lines during the prior 4 months. Among checklists present in the chart (*n* = 364), 83% were completed (no required fields left blank).

### Outcome Measures

#### Healthcare-Associated Bloodstream Infections

**Figure 3:**
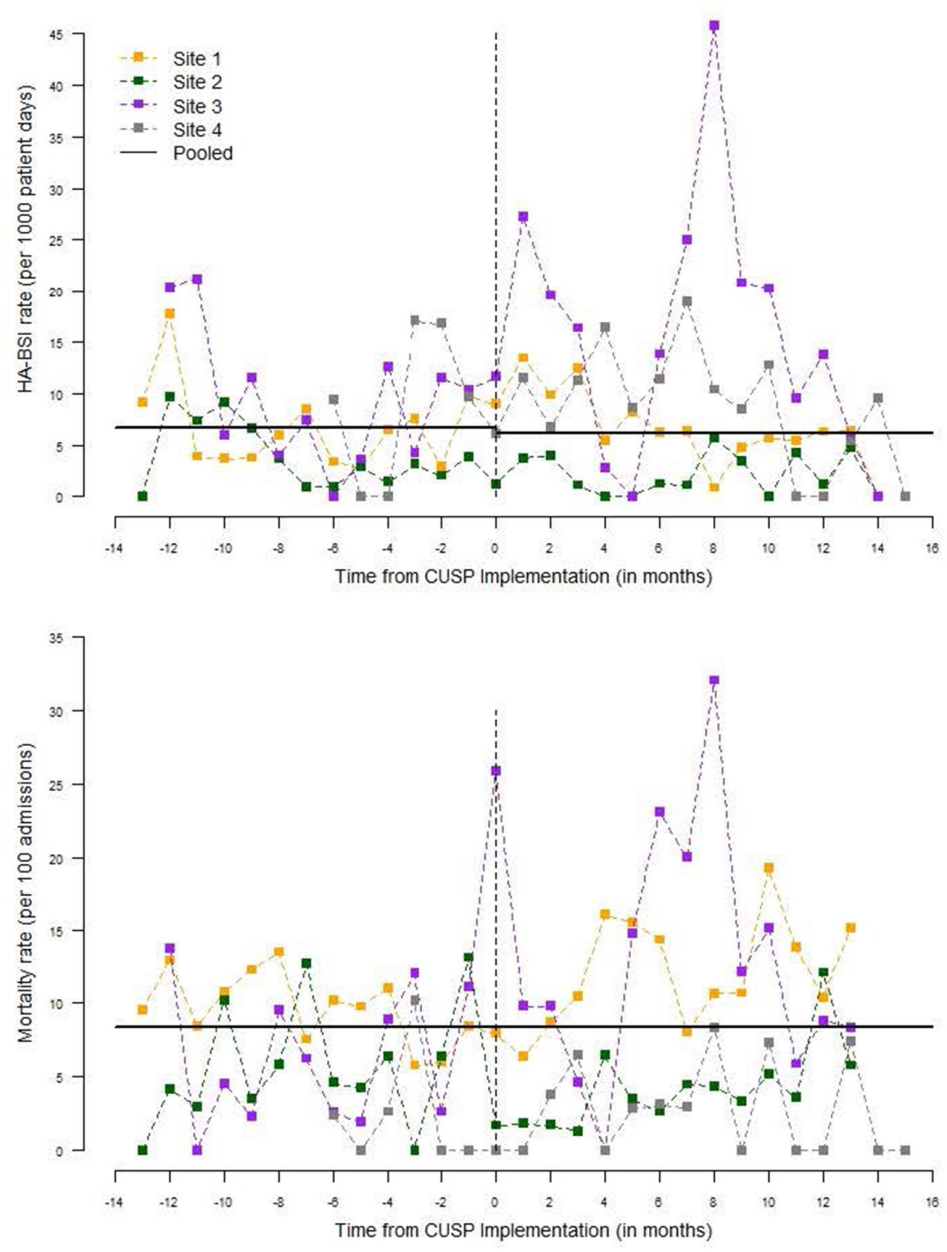
Healthcare-associated bloodstream infection rate and all-cause mortality by site and month. The monthly HA-BSI was expressed as cases per 1,000 patient-days. All-cause mortality was expressed as deaths per 100 admissions among neonates admitted for at least 3 days. The pooled estimates are based on a Poisson regression model for the monthly rate of HA-BSI or mortality as a function of post- vs. pre-CUSP implementation. CUSP, Comprehensive Unit-based Safety Program; HA-BSI, healthcare-associated bloodstream infection.

During the baseline period, there were 202 HA-BSI cases, with an HA-BSI rate of 5.99 per 1,000 patient-days (Figure 3). During the post-intervention period, there were 251 HA-BSI cases, with an HA-BSI rate of 6.40 per 1,000 patient-days. Overall, there was no statistically significant change in the monthly HA-BSI rate from baseline to the post-intervention period, with a relative rate (RR) of 0.97 [95% confidence interval (CI) 0.92–1.03] (Figure 3). HA-BSI rates demonstrated seasonality with increased rates over the monsoon period (June-September), which coincided with the start of the post-intervention period. There was no change in monthly HA-BSI rates for sites 1 and 4 (Table 3). Site 2 demonstrated a decrease in monthly HA-BSI rate from baseline to post-intervention (RR 0.52; 95% CI 0.32–0.84), whereas site 3 had an increased monthly HA-BSI rate post-intervention (RR 1.72; 95% CI 1.08–2.74).

**Table 3:** Healthcare-associated bloodstream infection rate and all-cause mortality at baseline and post-intervention, by site.

**HA-BSI**	**HA-BSI cases**	**Patient-days**	**HA-BSI rate per 1,000 patient-days**	**RR (95% CI)**
Site 1
Baseline	114	17,743	6.4	Ref
Post-intervention	142	19,953	7.1	1.11 (0.87–1.42)
Site 2
Baseline	47	11,452	4.1	Ref
Post-intervention	26	12,152	2.1	**0.52 (0.32**–**0.84)**
Site 3
Baseline	28	3,133	8.9	Ref
Post-intervention	49	3,188	15.4	**1.72 (1.08**–**2.74)**
Site 4
Baseline	13	1,369	9.50	Ref
Post-intervention	34	3,924	8.66	0.91 (0.48–1.73)
**All-cause mortality**	**Deaths**	**Admissions**	**Deaths per 100 admissions**	**RR (95% CI)**
Site 1
Baseline	214	2,192	9.76	Ref
Post-intervention	220	1,850	11.89	1.21 (1.00–1.46)
Site 2
Baseline	47	787	5.97	Ref
Post-intervention	35	839	4.17	0.68 (0.44–1.05)
Site 3
Baseline	30	481	6.24	Ref
Post-intervention	61	488	12.50	**2.00 (1.29**–**3.10)**
Site 4
Baseline	7	245	2.86	Ref
Post-intervention	15	552	2.72	0.95 (0.39–2.34)

*Healthcare-associated bloodstream infections were defined as positive blood cultures with a known neonatal bacterial or fungal pathogen occurring on hospital day 3 or greater. Patient-days at risk were calculated as patient-days from hospital day 3 until NICU exit. All-cause mortality was defined as deaths per 100 admissions among neonates admitted for at least 3 days. CI, confidence interval; HA-BSI, healthcare-associated bloodstream infection; RR, relative rate. Bolded values reach statistical significance*.

#### All-Cause Mortality

Monthly all-cause mortality was unchanged from baseline to the post-intervention period across the four sites (RR 1.00, 95% CI 0.69–1.46) (Figure 3). Sites 1, 2, and 4 had no change in monthly all-cause mortality, whereas site 3 had an increase from baseline to post-intervention (RR 2.00; 95% CI 1.29–3.10) (Table 3).

#### Patient Safety Culture

##### Respondent Characteristics

The baseline HSOPS was completed by 182 respondents (response rate 85.8%). The follow-up HSOPS was completed by 212 respondents (response rate 99.1%) (Table 4). The majority of respondents were nurses, 163 (89.6%) at baseline and 182 (85.0%) at follow-up. All respondents identified as having direct patient contact.

**Table 4:** Hospital Survey on Patient Safety Culture respondents by healthcare worker role at baseline and follow-up, by site.

	**Nurse**	**Physician**	**Other**	**Unknown**	**Total**
	***n* = 345**	***n* = 47**	***n* = 1**	***n* = 3**	***n* = 396**
Site 1
Baseline	64	0	0	0	64
Follow-up	72	1	0	1	74
Site 2
Baseline	53	1	0	0	54
Follow-up	58	6	1	2	67
Site 3
Baseline	19	6	0	0	25
Follow-up	15	12	0	0	27
Site 4
Baseline	27	12	0	0	39
Follow-up	37	9	0	0	46
Total
Baseline	163	19	0	0	182
Follow-up	182	28	1	3	214

*The HSOPS was completed anonymously by HCWs at baseline and follow-up. Respondents could select from multiple choice options of staff positions or provide a free text response. Categories of physicians (attending physician, resident physician, etc.) and nurses (charge/head nurse, nurse, nursing student, etc.) were collapsed. Only one respondent identified as an HCW other than physician or nurse. Three respondents did not provide their staff position. All respondents described themselves as having direct patient contact. No administrators completed the survey. HCW, healthcare worker; HSOPS, Hospital Survey on Patient Safety Culture*.

##### Survey Results

High-scoring dimensions at baseline included teamwork within units (mean PPS 81.7% across all sites), supervisor/manager expectations and actions promoting patient safety (78.0%), organizational learning—continuous improvement (85.9%), and patient safety grade (81.8%). Improvements in mean PPS were seen in key dimensions of patient safety culture, with the largest increases in management support for patient safety (+7.6%), teamwork within units (+5.3%), management support for patient safety (+4.7%), and supervisor/manager expectations and actions promoting patient safety (+3.4%). Notably, there was a decrease in communication openness (−10.8%) and patient safety grade (−5.2%).

Survey responses varied by site (Table 5; Supplementary Figures 1a–m). For site 1, the largest gains were in frequency of event reporting (+30.3%), teamwork within units (+15.7%), teamwork across units (+11.9%), and management support for patient safety (+11.1%). For site 2, the composite domains with the greatest increases were non-punitive response to errors (+8.8%), organizational learning—continuous improvement (+8.4%), management support for patient safety (+6.6%), and staffing (+4.2%). For site 3, the largest gains were in supervisor/manager expectations & actions promoting patient safety (+11.7%), teamwork within units (+10.8%), non-punitive response to errors (+8.1%), and handoffs and transitions (+6.2%). For site 4, the greatest improvement was seen in management support for patient safety (+9.9%), patient safety grade (+4.9%), staffing (+3.8%), and organizational learning—continuous improvement (+2.8%).

**Table 5:** Mean percent positive scores by composite dimension, baseline and follow-up Hospital Survey on Patient Safety Culture by site.

	**Baseline (%)**	**Follow-up (%)**	**Difference (%)**	**95% CI (%)**
**Site 1**
Teamwork within units	58.6	74.3	**+15.7**	**(5.9, 25.7)**
Supervisor/manager expectations and actions promoting patient safety	77.6	82.1	+4.5	(−4.2, 13.6)
Organizational learning—continuous improvement	91.1	92.2	+1.1	(−4.7, 6.8)
Management support for patient safety	55.6	66.7	**+11.1**	**(9.4, 21.2)**
Perceptions of patient safety	58.6	56.1	−2.5	(−10.7, 4.9)
Feedback and communication about error	57.3	64.0	+6.7	(−2.8, 17.2)
Communication openness	74.7	62.2	−12.5	(−21.9, −2.8)
Frequency of event reporting	32.3	62.6	**+30.3**	**(16.9, 43.3)**
Teamwork across units	53.0	64.9	**+11.9**	**(1.8, 22.2)**
Staffing	23.8	27.7	+3.9	(−1.5, 9.0)
Handoffs and transitions	53.4	54.8	+1.4	(−8.8, 11.9)
Non-punitive response to errors	28.1	28.4	+0.3	(−9.4, 10.8)
Patient safety grade	70.3	67.6	−2.7	(−17.7, 13.4)
**Site 2**
Teamwork within units	95.4	92.2	−3.2	(−7.5, 1.4)
Supervisor/manager expectations and actions promoting patient safety	85.6	86.6	+1.0	(−6.5, 8.1)
Organizational learning—continuous improvement	81.5	89.9	+8.4	(−0.1, 16.2)
Management support for patient safety	75.3	81.9	+6.6	(−2.7, 16.4)
Perceptions of patient safety	60.7	57.5	−3.2	(−10.7, 4.5)
Feedback and communication about error	73.8	73.1	−0.7	(−11.3, 9.5)
Communication openness	79.0	73.9	−5.1	(−13.9, 4.1)
Frequency of event reporting	66.7	64.2	−2.5	(−15.2, 10.1)
Teamwork across units	78.1	81.7	+3.6	(−4.2, 11.7)
Staffing	38.4	42.6	+4.2	(−2.1, 10.6)
Handoffs and transitions	68.1	62.1	−6.0	(−17.4, 5.6)
Non-punitive response to errors	39.5	48.3	+8.8	(−0.9, 17.8)
Patient safety grade	92.6	94.0	+1.4	(−7.1, 10.3)
**Site 3**
Teamwork within units	80.0	90.8	+10.8	(−2.8, 25.2)
Supervisor/manager expectations and actions promoting patient safety	67.0	78.7	+11.7	(−3.0, 26.6)
Organizational learning—continuous improvement	81.3	87.7	+6.4	(−5.5, 18.6)
Management support for patient safety	61.3	64.2	+1.9	(−12.8, 18.1)
Perceptions of patient safety	49.0	54.7	+5.7	(−7.2, 17.0)
Feedback and communication about error	56.0	42.5	−13.5	(−32.6, 6.3)
Communication openness	58.7	45.7	−13.0	(−32.9, 8.0)
Frequency of event reporting	52.0	34.6	−17.4	(−36.6, 2.5)
Teamwork across units	67.0	53.7	−13.3	(−26.0, 7.4)
Staffing	29.0	32.0	+3.0	(−5.5, 10.7)
Handoffs and transitions	52.8	59.0	+6.2	(−10.6, 24.0)
Non-punitive response to errors	40.0	48.1	+8.1	(−9.5, 27.1)
Patient safety grade	80.0	55.6	−24.4	(−47.9, 1.8)
**Site 4**
Teamwork within units	92.9	90.8	−2.1	(−8.7, 5.2)
Supervisor/manager expectations and actions promoting patient safety	81.9	78.2	−3.7	(−14.2, 7.0)
Organizational learning—continuous improvement	89.8	92.6	+2.8	(−4.3, 10.9)
Management support for patient safety	71.2	81.1	+9.9	(−1.6, 19.7)
Perceptions of patient safety	66.1	67.2	+1.1	(−8.0, 9.8)
Feedback and communication about error	67.2	60.3	−6.9	(−20.2, 6.3)
Communication openness	66.6	54.3	−12.3	(−26.0, 2.6)
Frequency of event reporting	57.8	55.8	−2.0	(−19.6, 14.3)
Teamwork across units	77.5	77.7	+0.2	(−9.4, 9.5)
Staffing	28.8	32.6	+3.8	(−5.7, 12.7)
Handoffs and transitions	68.9	64.7	−4.2	(−19.1, 9.9)
Non-punitive response to errors	46.1	38.0	−8.1	(−23.1, 7.3)
Patient safety grade	84.2	89.1	+4.9	(−9.0, 19.8)

*The HSOPS consists of 42 items into 12 composite domains that assess elements of patient safety culture using a Likert response scale. PPS by item were calculated by dichotomizing responses and reverse coding for negative items. Mean PPS for composite domains were calculated by averaging PPS across items included in each domain. Patient safety grade was determined by calculating mean response to a single item. HSOPS, Hospital Survey on Patient Safety Culture; PPS, percent positive score. Bolded values reach statistical significance*.

In exploring overall trends, all four sites demonstrated improvements in the following composite domains: organizational learning—continuous improvement, management support for patient safety, and staffing. Communication openness was the only composite domain that had decreased follow-up scores at all sites.

#### Workload

Workload increased across all six domains from baseline to post-intervention periods (Table 6). Mental demand increased from a mean of 7.44 ± 4.35 to 11.16 ± 5.20 (*p* < 0.0001), physical demand from 7.57 ± 4.41 to 11.95 ± 4.85 (*p* < 0.0001), temporal demand from 7.55 ± 4.47 to 10.60 ± 4.25 (*p* < 0.0001), effort from 8.15 ± 4.06 to 11.10 ± 4.37 (*p* < 0.0001), performance from 10.07 ± 12.15 (*p* < 0.0001), and frustration from 5.50 ± 2.94 to 8.11 ± 4.45 (*p* < 0.0001). Overall workload increased from 46.28 ± 16.97 to 65.07 ± 19.05 (*p* < 0.0001).

**Table 6:** Workload among neonatal intensive care unit staff at baseline and post-intervention, as measured by NASA-TLX.

**Item**	**Baseline, mean ± SD (range)**	**Post-intervention, mean ± SD (range)**	***P*-value**
**Site 1**
Mental	8.78 ± 4.89	12.2 ± 4.73	**0.0001**
Physical	9.54 ± 4.87	13.06 ± 4.97	**<0.0001**
Temporal	8.89 ± 4.42	11.23 ± 4.09	**0.0016**
Effort	9.75 ± 4.66	11.81 ± 3.97	**0.0059**
Performance	10.60 ± 4.17	11.00 ± 4.60	0.5952
Frustration	6.27 ± 3.28	8.91 ± 4.32	**0.0001**
Overall	53.82 ± 16.67	68.21 ± 18.89	**<0.0001**
**Site 2**
Mental	6.60 ± 4.18	9.65 ± 5.14	**0.0006**
Physical	6.41 ± 3.90	9.91 ± 4.62	**<0.0001**
Temporal	8.61 ± 4.54	8.69 ± 3.59	0.9189
Effort	7.00 ± 3.45	8.97 ± 3.64	**0.0030**
Performance	8.97 ± 4.95	12.73 ± 4.36	**<0.0001**
Frustration	5.35 ± 2.74	6.79 ± 3.69	**0.0186**
Overall	42.95 ± 16.79	56.74 ± 16.21	**<0.0001**
**Site 3**
Mental	7.19 ± 4.10	14.61 ± 4.57	**<0.0001**
Physical	6.48 ± 3.54	15.30 ± 3.66	**<0.0001**
Temporal	5.93 ± 3.01	14.11 ± 4.07	**<0.0001**
Effort	7.92 ± 3.15	15.51 ± 4.35	**<0.0001**
Performance	10.72 ± 4.28	9.44 ± 6.27	0.3991
Frustration	5.48 ± 2.45	12.37 ± 5.35	**<0.0001**
Overall	43.72 ± 15.22	81.34 ± 15.66	**<0.0001**
**Site 4**
Mental	6.57 ± 3.30	9.66 ± 5.12	**0.0018**
Physical	6.67 ± 3.74	11.17 ± 4.09	**<0.0001**
Temporal	4.91 ± 3.85	10.32 ± 4.03	**<0.0001**
Effort	7.26 ± 3.57	10.47 ± 3.83	**0.0002**
Performance	10.31 ± 4.06	14.76 ± 3.93	**<0.0001**
Frustration	4.46 ± 2.62	6.24 ± 2.97	**0.0048**
Overall	40.18 ± 14.74	62.61 ± 18.11	**<0.0001**
**All sites**
Mental	7.44 ± 4.35	11.16 ± 5.20	**<0.0001**
Physical	7.57 ± 4.41	11.95 ± 4.85	**<0.0001**
Temporal	7.55 ± 4.47	10.60 ± 4.25	**<0.0001**
Effort	8.15 ± 4.06	11.10 ± 4.37	**<0.0001**
Performance	10.07 ± 4.43	12.15 ± 4.91	**<0.0001**
Frustration	5.50 ± 2.94	8.11 ± 4.45	**<0.0001**
Overall	46.28 ± 16.97	65.07 ± 19.05	**<0.0001**

*The NASA-TLX was completed at baseline and post-intervention to assess workload across six domains on a 20-point continuous scale. Overall workload was calculated by summing scores for the six domains at baseline and post-intervention. NASA-TLX, National Aeronautics and Space Administration Task Load Index. Bolded values reach statistical significance*.

## Discussion

Our study fostered the creation of multidisciplinary CUSP teams that collaboratively selected and implemented interventions to improve IPC practices in three focus areas, hand hygiene, aseptic technique for invasive procedures, and medication and IV fluid preparation and administration. CUSP enabled sites to pursue multimodal IPC improvement strategies that included a focus on patient safety culture, as championed by the WHO (29). This intervention led to an increase in hand hygiene but did not reduce HA-BSI or all-cause mortality during the study period. While one site did have a reduction in HA-BSI rate, another site experienced an increase in both HA-BSI and mortality, which was largely driven by an outbreak that occurred during the post-intervention period. The CUSP team was instrumental in developing an outbreak response and focusing on appropriate IPC interventions, including a heightened focus on the importance of hand hygiene.

CUSP did lead to process improvements known to reduce infection risk, including a marked improvement in hand hygiene and successful implementation of a central line insertion checklist. The success of CUSP implementation can also be measured by the observed culture change and practice changes. Over the course of the study period, it was evident that nurses became empowered to speak up in front of leadership and advocate for patient safety and nurses attending CUSP meetings increasingly felt ownership and pride in the interventions they were leading. Furthermore, monthly coaching calls facilitated sharing of strategies and dissemination of interventions among sites, serving as a new forum for collaboration among NICUs facing similar challenges.

All sites demonstrated improved hand hygiene over the course of the study period. Implementing a program of hand hygiene compliance monitoring along with feedback of data to CUSP teams and unit staff, constituted a powerful intervention. While all sites previously employed some form of hand hygiene compliance monitoring by infection control, there was no consistent feedback or sharing of data with unit-level staff prior to CUSP implementation. Monthly hand hygiene data provided direct feedback to CUSP teams regarding the impact of their interventions and provided an opportunity to tailor interventions to specific HCW roles and moments of hand hygiene as needed. At the end of the study, sites committed to continue hand hygiene compliance monitoring and feedback of data.

Implementation of a central line insertion checklist are evidence-based practices that represented a paradigm shift in patient safety for these NICUs (30, 31). Prior to CUSP, none of the participant NICUs used pre-operative or pre-procedural checklists. Sites readily adapted its use into daily medical practice, with gradual increases in appropriate completion of checklists used. With checklist implementation, the importance of having an observer or assistant present for central line insertion was highlighted, which can be challenging in a resource-limited setting. The checklist also empowered nurses or other HCWs serving in the observer/assistant role to intervene if steps of appropriate aseptic technique were not followed. By site request, a central line maintenance audit tool was created by the study team, though its completion was not audited by study staff. Both the insertion checklist and the maintenance audit tool fit well into the existing healthcare system at our four sites, with checklists used for a variety of other indications.

During our baseline IPC assessments, we noted opportunities for improvement at all sites for practices related to medication and IV fluid preparation and administration. In a resource-limited setting, challenges to injection safety include reliance on multi-dose vials, reuse of single-dose vials, and large stock bottles of IV fluid solutions and topical antiseptics including alcohol and betadine, as well as preparation of medications and IV fluids within the immediate patient care area (32, 33). While it was not possible to transition to exclusive use of single-dose vials and eliminate use of large stock bottles, CUSP teams focused on how to improve injection safety by including IPC considerations in adapting existing workflow.

Multiple studies have demonstrated that improving patient safety culture across HSOPS domains is associated with lower HAI rates (34, 35). Sites demonstrated gains in key HSOPS domains of patient safety, including teamwork within units, supervisor/manager expectations and actions promoting patient safety, organizational learning—continuous improvement, and management support for patient safety. In considering the elements of the CUSP intervention, these are the domains in which one would expect improvement. Most gains did not reach statistical significance which likely reflects a relatively fixed sample size of HCWs employed within each unit. Our response rates for both baseline and follow-up surveys were excellent and we hope to explore whether these trends continue and assess generalizability of our findings by recruiting more sites.

We did not expect to improve other domains, such as staffing and teamwork across units, which were outside of the scope of our intervention. The notable decrease in communication openness across all sites should be explored further and addressed in moving CUSP forward. We did not see a significant improvement in patient safety grade, which consists of a single item asking respondents to assign a letter grade to their unit's patient safety. It is not surprising that respondents are more critical of patient safety after an intervention that raises knowledge and awareness of IPC practices and their impact on patient safety.

As measured by NASA-TLX across six domains, workload increased from baseline to the post-intervention period. To our knowledge, NASA-TLX assessment of workload has not previously been used in the context of CUSP implementation. Tubbs-Cooley et al. previously measured overall workload among neonatal, pediatric, and adult intensive care nurses using NASA-TLX; among NICU nurses who participated in this multi-center cross-sectional study, the mean overall workload was similar to that reported on our study, roughly midway between our baseline and post-intervention scores (36). CUSP implementation required a comprehensive unit-level shift in the approach to IPC and patient safety. While CUSP activities were led by the physician and nurse champions in conjunction with the CUSP team, selected interventions within the three focus areas required participation and commitment by all unit staff, especially those providing direct patient care. CUSP may have contributed to the increase in workload seen over the course of this study, though other unmeasured factors such as staffing changes or more complex patient load could have also contributed. Effect of CUSP activities on perceived workload should be monitored closely, given the association of increased NASA-TLX scores with HCW burnout (37). Furthermore, a single-center study in a United States NICU described an association between increased nursing workload with missed nursing care (38). CUSP should prioritize interventions that lighten workload by making work more efficient and less burdensome, though advocating for improved staffing may also be a critical CUSP activity.

Our intervention coincided with an increased focus on patient safety by the Indian Ministry of Health. Multiple sites underwent accreditation or government official visits during the study period. Our intervention aligned well with this mission, especially with regard to monitoring of hand hygiene compliance and use of a central line insertion checklist. Senior executive support of CUSP-driven interventions led to spread beyond the NICU, with adaptation of some interventions throughout the hospital.

Strengths of this study include use of a multidisciplinary team that worked together in an iterative process that allows for learning and is firmly grounded in creation of local patient safety culture to reduce HAI risk. This study demonstrated the ready adaptation of an existing toolkit that has proven success to a resource-limited setting. The selected focus areas included high-yield IPC practices, optimization of which has been linked to reduction of HA-BSI in a variety of healthcare settings. Site-driven selection of interventions, rather than a prepackaged IPC bundle, yielded locally appropriate solutions to IPC gaps that are more likely to be sustainable than financially burdensome interventions.

Limitations of this study include its duration and small sample size. A 16-month study period limited our capacity to assess the impact of the intervention on HA-BSI rates and mortality, especially given seasonality of infections in this setting, with higher rates seen during monsoon season. Additionally, the final focus area, medication and IV fluid preparation and administration, was introduced only 3 months prior to study end, limiting our capacity to assess the impact of selected interventions on outcomes of interest. However, improved hand hygiene and gains seen in patient safety culture are promising measures of success of this intervention. Though the advent of the coronavirus disease 2019 pandemic disrupted CUSP activities, sites have committed to sustaining the CUSP intervention. We intend to complete a follow-up assessment of the impact of CUSP, which will provide important information about CUSP sustainability in this setting.

CUSP is a promising multimodal strategy for healthcare facilities in resource-limited settings that encompasses key aspects of the WHO's IPC improvement strategy, including culture of safety. Our study outlines an approach to CUSP that can be readily adapted to NICUs in an LMIC setting and is feasible. Next steps include assessment of sustainability and generalizability of our findings.

## Data Availability Statement

The raw data supporting the conclusions of this article will be made available by the authors, without undue reservation.

## Ethics Statement

The studies involving human participants were reviewed and approved by Johns Hopkins Medicine Institutional Review Board, Byramjee-Jeejeebhoy Government Medical College Ethics Committee, Dr. D. Y. Patil Medical College Ethics Committee, King Edward Memorial Hospital Ethics Committee, Bharati Vidyapeeth Medical College Ethics Committee. The patients/participants provided their written informed consent to participate in this study.

## Author Contributions

BR and AKa supported data collection. JJ and EC analyzed the data. JJ drafted the initial manuscript. All authors conceptualized and designed the study and contributed to the manuscript revision.

## Funding

This study was supported by the United States Centers for Disease Control and Prevention (Safe Healthcare, Epidemiology, and Prevention Research Development Program Domain 7, Contract 200-2016-91781). JJ receives support from the National Institutes of Health (K23HD100594). MR receives support from the National Institutes of Health (UM1AI104681). VM receives support from the National Institutes of Health (UM1AI069465-13, 1R01AI43748-01A1). AG receives support from the National Institutes of Health (UM1AI069465, UM1AI068632, and UM1AI068636). AM receives support from the National Institutes of Health (K24AI141580).

## Conflict of Interest

The authors declare that the research was conducted in the absence of any commercial or financial relationships that could be construed as a potential conflict of interest.

## Publisher's Note

All claims expressed in this article are solely those of the authors and do not necessarily represent those of their affiliated organizations, or those of the publisher, the editors and the reviewers. Any product that may be evaluated in this article, or claim that may be made by its manufacturer, is not guaranteed or endorsed by the publisher.
