# Supplementary material for: Implementation of the Comprehensive Unit-Based Safety Program to Improve Infection Prevention and Control Practices in Four Neonatal Intensive Care Units in Pune, India
Source: Front Pediatr. 2022 Jan 6;9:794637. doi: 10.3389/fped.2021.794637 (PMC8772032; doi:10.3389/fped.2021.794637)
Supplement: Supplementary file 1 [file Data_Sheet_1.PDF]

Supplement. Supplemental Tables, Figures, and CUSP Tools

Supplemental Table 1. Staff Safety Assessment questions by focus area. For each focus area, unit-based staff completed the two-question SSA to identify perceived challenges and potential interventions to improve IPC practices within each focus area. SSA responses guided CUSP team selection of multimodal interventions within each focus area. Abbreviations: CUSP – Comprehensive Unit-based Safety Program; IPC – infection prevention and control; IV – intravenous; SSA – Staff Safety Assessment.

**Focus area**	**SSA questions**
Hand hygiene	Describe the challenges in achieving the highest standard of hand hygiene practices in your NICU.<br>Describe what you think can be done to overcome these challenges.
Aseptic technique for invasive procedures	What will be the next cause of lack of aseptic technique during a procedure on your unit?<br>Describe what you think can be done to prevent or minimize this harm.
Medication and IV fluid preparation	What will be the next cause of unsafe injection practices on your unit?<br>Describe what you think can be done to prevent or minimize this harm.

Supplemental Table 2. Hospital Survey on Patient Safety Culture items and composite domains. The HSOPS consists of 42 items organized into 12 composite domains and a patient safety grade. Responses are provided on a Likert scale, consisting of (1) Strongly Disagree, Disagree, Neither Agree nor Disagree, Agree, Strongly Agree or (2) Never, Rarely, Sometimes, Most of the time, Always. Negatively worded questions are reverse coded for calculation of PPS by item and composite domains. Abbreviations: HSOPS – Hospital Survey on Patient Safety Culture; PPS – percent positive score.

Composite Domain	Items
Teamwork within units	A1. People support one another in this unit.
	A3. When a lot of work needs to be done quickly, we work together as a team to get the work done.
	A4. In this unit, people treat each other with respect.
	A11. When one area in this unit gets really busy, others help out.
Supervisor/manager expectations & actions promoting patient safety	B1. My supervisor/manager says a good word when he/she sees a job done according to established patient safety procedures.
	B2. My supervisor/manager seriously considers staff suggestions for improving patient safety.
	B3. Whenever pressure builds up, my supervisor/manager wants us to work faster, even if it means taking shortcuts. (negatively worded)
	B4. My supervisor/manager overlooks patient safety programs that happen over and over. (negatively worded)
Organizational learning – continuous improvement	A6. We are actively doing things to improve patient safety.
	A9. Mistakes have led to positive changes here.
	A13. After we make changes to improve patient safety, we evaluate their effectiveness.
Management support for patient safety	F1. Hospital management provides a work climate that promotes patient safety.
	F8. The actions of hospital management show that patient safety is a top priority.
	F9. Hospital management seems interested in patient safety only after an adverse event happens. (negatively worded)
Overall perceptions of patient safety	A15. Patient safety is never sacrificed to get more work done.
	A18. Our procedures and systems are good at preventing errors from happening.
	A10. It is just by chance that more serious mistakes don’t happen around here. (negatively worded)
	A17. We have patient safety problems in this unit. (negatively worded)
Feedback & communication about error	C1. We are given feedback about changes put into place based on event reports.
	C3. We are informed about errors that happen in this unit.
	C5. In this unit, we discuss ways to prevent errors from happening again.
Communication openness	C2. Staff will freely speak up if they see something that may negatively affect patient care.

	C4. Staff feel free to question the decisions or actions of those with more authority.<br><br>C6. Staff are afraid to ask questions when something does not seem right. (negatively worded)
Frequency of event reporting	D1. When a mistake is made, but is caught and corrected before affecting the patient, how often is this reported?<br><br>D2. When a mistake is made, but has no potential to harm the patient, how often is this reported?<br><br>D3. When a mistake is made that could harm the patient, but does not, how often is this reported?
Teamwork across units	F4. There is good cooperation among hospital units that need to work together.<br><br>F10. Hospital units work well together to provide the best care for patients.<br><br>F2. Hospital units do not coordinate well with each other. (negatively worded)<br><br>F6. It is often unpleasant to work with staff from other hospital units. (negatively worded)
Staffing	A2. We have enough staff to handle the workload.<br><br>A5. Staff in this unit work longer hours than is best for patient care. (negatively worded)<br><br>A7. We use more agency/temporary staff than is best for patient care. (negatively worded)<br><br>A14. We work in “crisis mode” trying to do too much, too quickly. (negatively worded)
Handoffs & transitions	F3. Things “fall between the cracks” when transferring patients from one unit to another. (negatively worded)<br><br>F5. Important patient care information is often lost during shift changes. (negatively worded)<br><br>F7. Problems often occur in the exchange of information across hospital units. (negatively worded)<br><br>F11. Shift changes are problematic for patients in this hospital. (negatively worded)
Nonpunitive response to errors	A8. Staff feel like their mistakes are held against them. (negatively worded)<br><br>A12. When an event is reported, it feels like the person is being written up, not the problem. (negatively worded)<br><br>A16. Staff worry that mistakes they make are kept in their personnel file. (negatively worded)
Patient safety grade	E1. Please give your work area/unit in this hospital an overall grade on patient safety.

Supplemental Figures 1a-m. Mean percent positive score by composite domain, Hospital Survey on Patient Safety Culture at baseline and difference at follow-up. The HSOPS consists of 42 items into 12 composite domains that assess elements of patient safety culture using a Likert response scale. PPS by item were calculated by dichotomizing responses and reverse coding for negative items. Mean PPS for composite domains were calculated by averaging PPS across items included in each domain. Each figure represents a domain and displays site baseline PPS (x-axis) and difference at follow-up (y-axis); the final figure represents the patient safety grade, which was determined by calculating mean response to a single item. Y-axis scale differs by figure based on range of mean PPS difference at follow-up. Abbreviations: HSOPS – Hospital Survey on Patient Safety Culture; PPS – percent positive score.

Supplemental Figure 1a. Teamwork within unit, percent positive score at baseline and difference at follow-up, by site

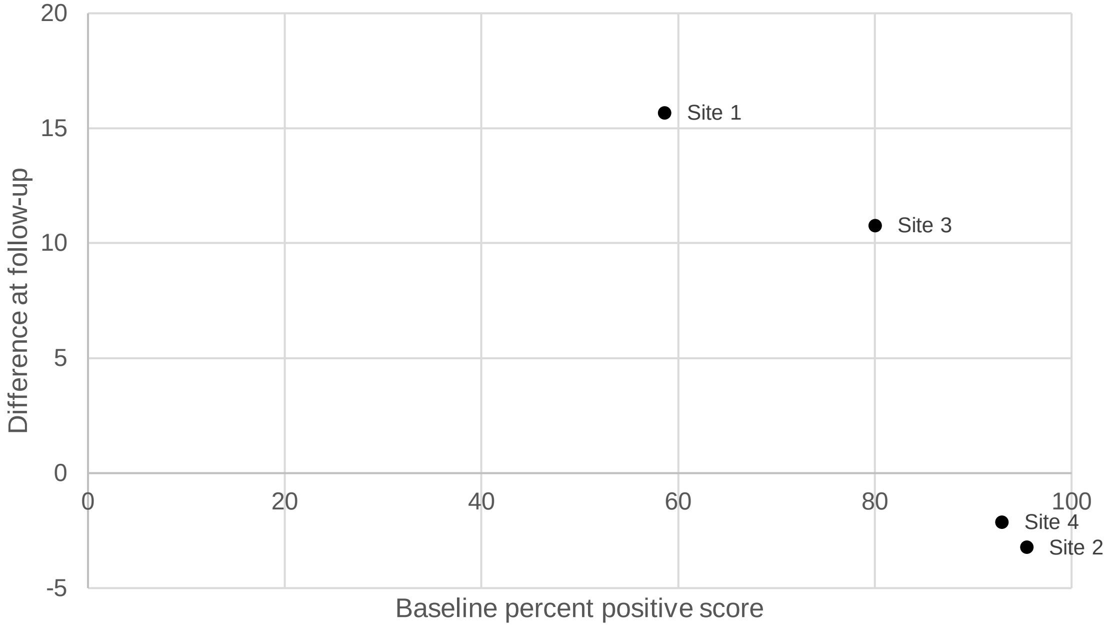

Supplemental Figure 1b. Supervisor/manager expectations & actions promoting patient safety, percent positive score at baseline and difference at follow-up, by site

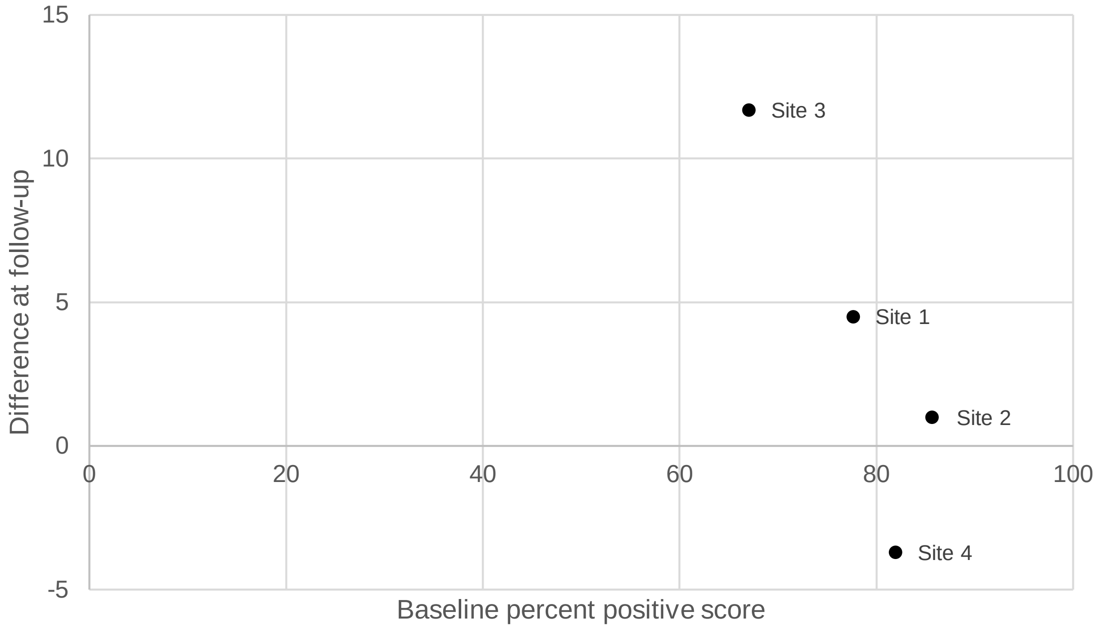

Supplemental Figure 1c. Organizational learning – continuous improvement, percent positive score at baseline and difference at follow-up, by site

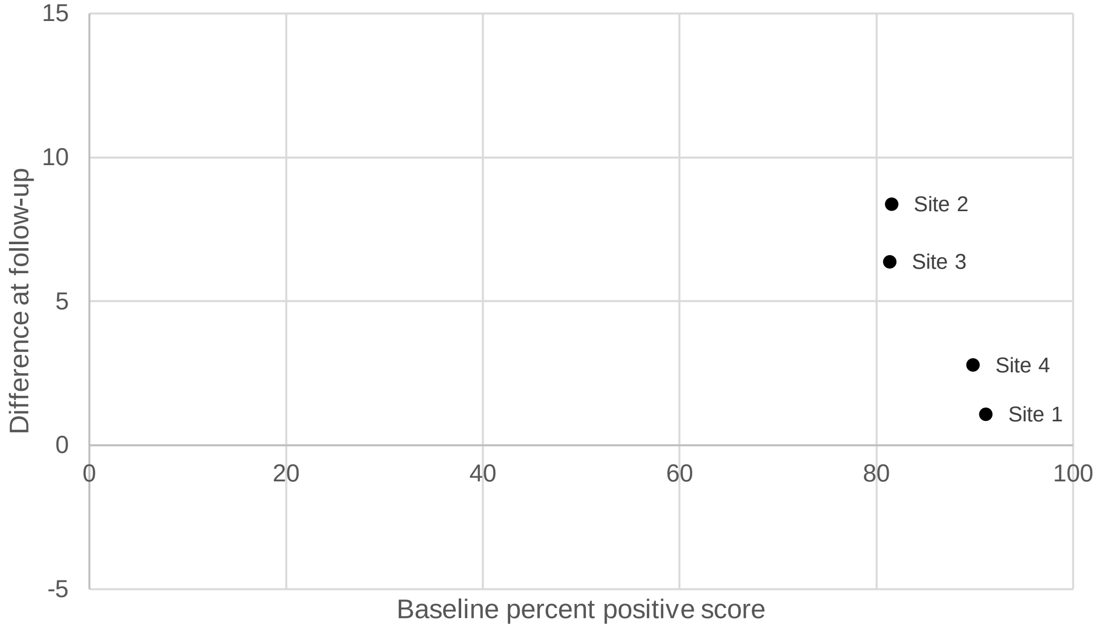

Supplemental Figure 1d. Management support for patient safety, percent positive score at baseline and difference at follow-up, by site

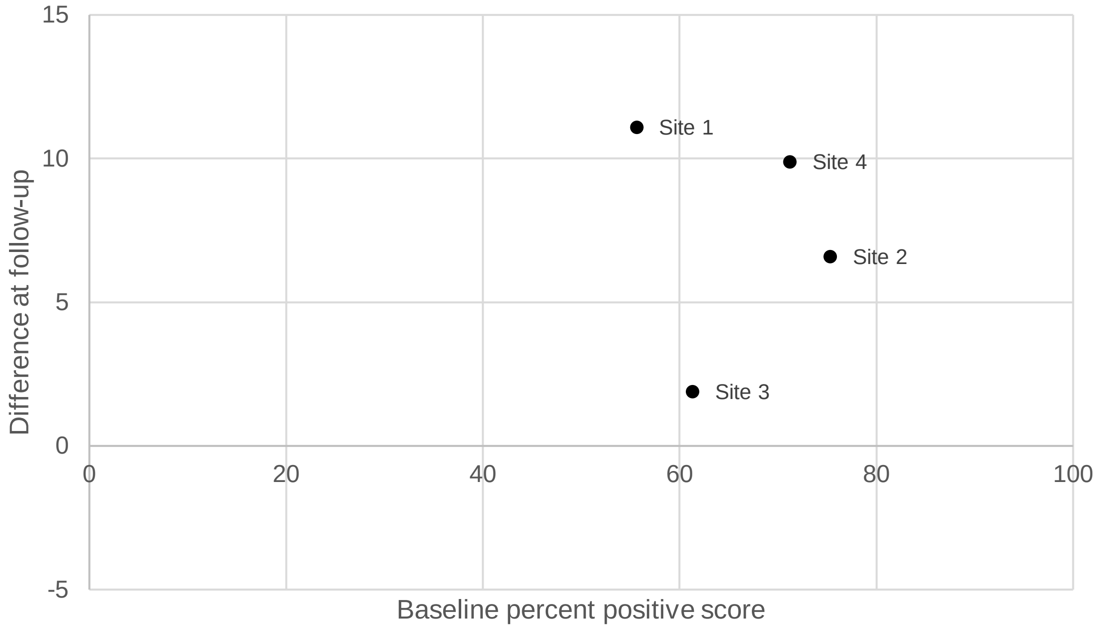

Supplemental Figure 1e. Perceptions of patient safety, percent positive score at baseline and difference at follow-up, by site

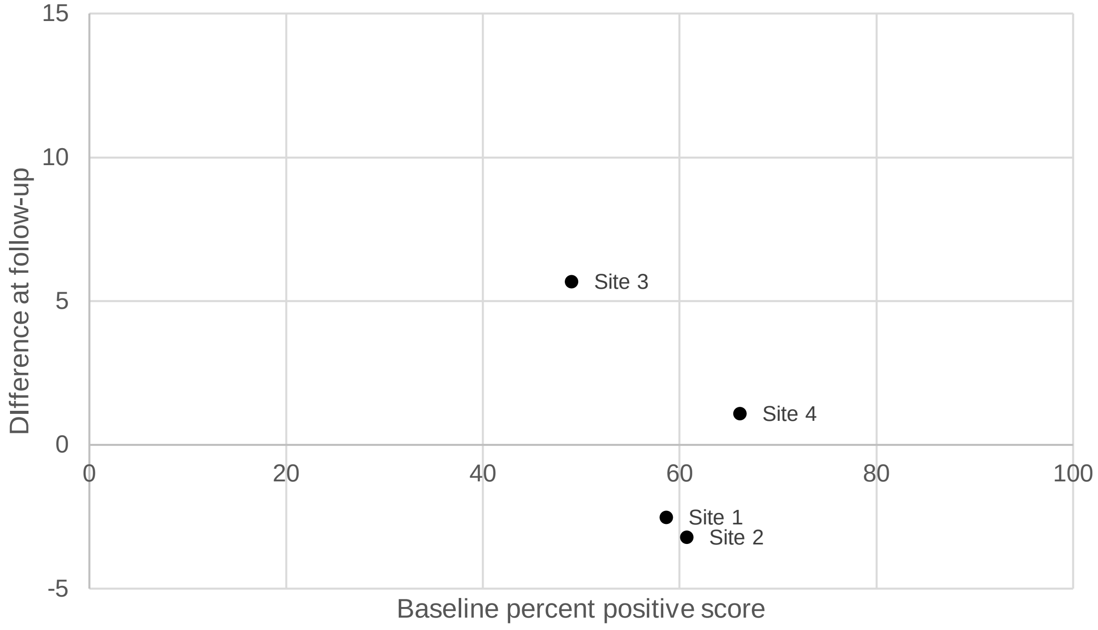

Supplemental Figure 1f. Feedback and communication about error, percent positive score at baseline and difference at follow-up, by site

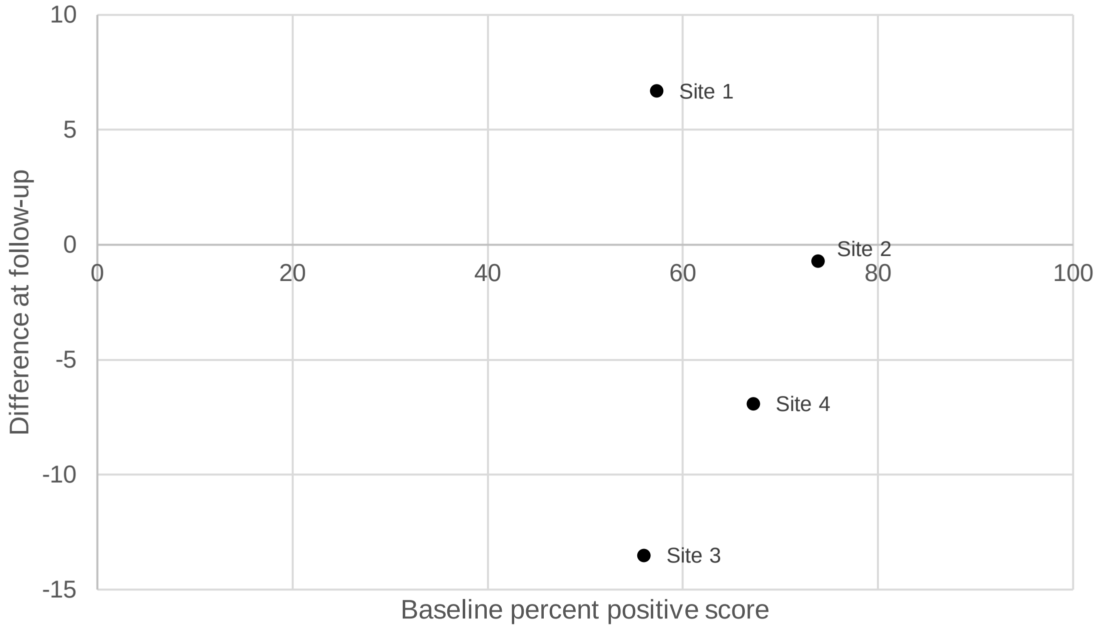

Supplemental Figure 1g. Communication openness, percent positive score at baseline and difference at follow-up, by site

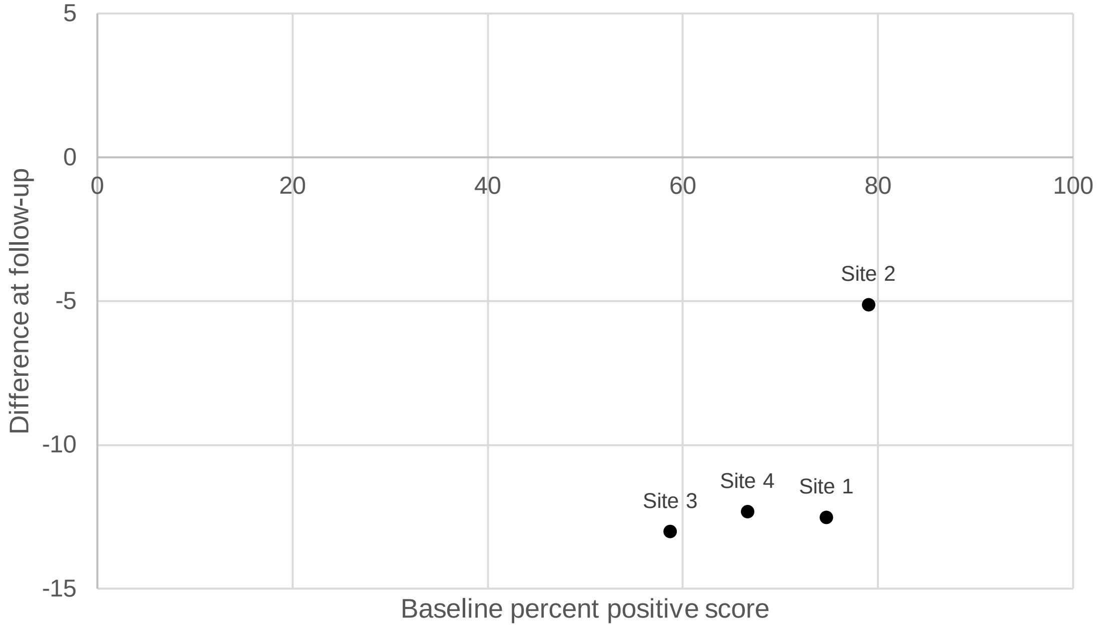

Supplemental Figure 1h. Frequency of event reporting, percent positive score at baseline and difference at follow-up, by site

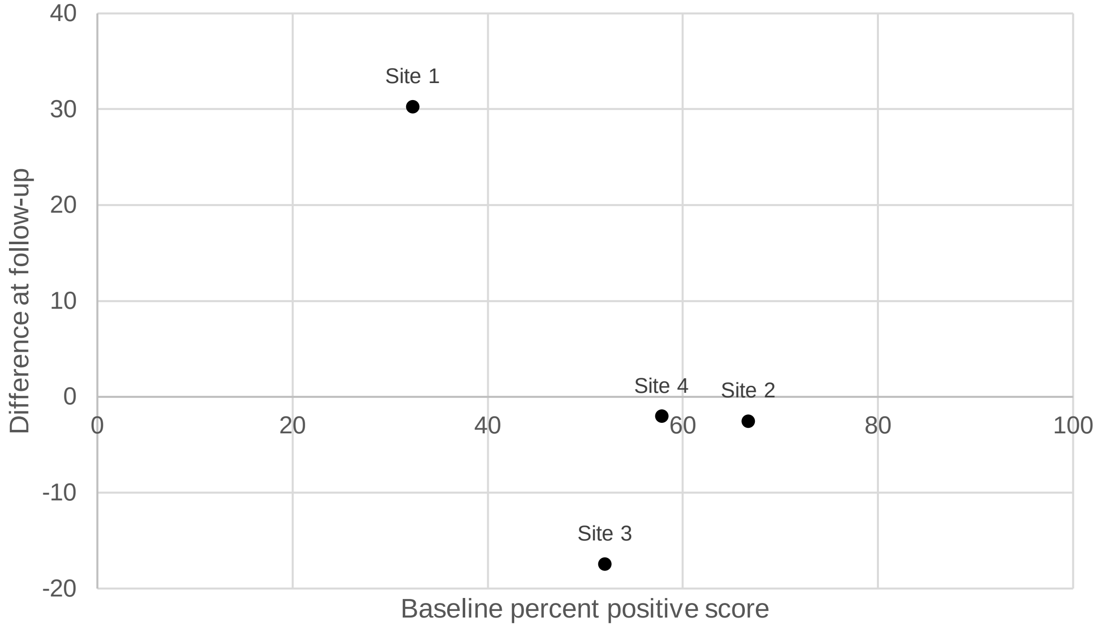

Supplemental Figure 1i. Teamwork across units, percent positive score at baseline and difference at follow-up, by site

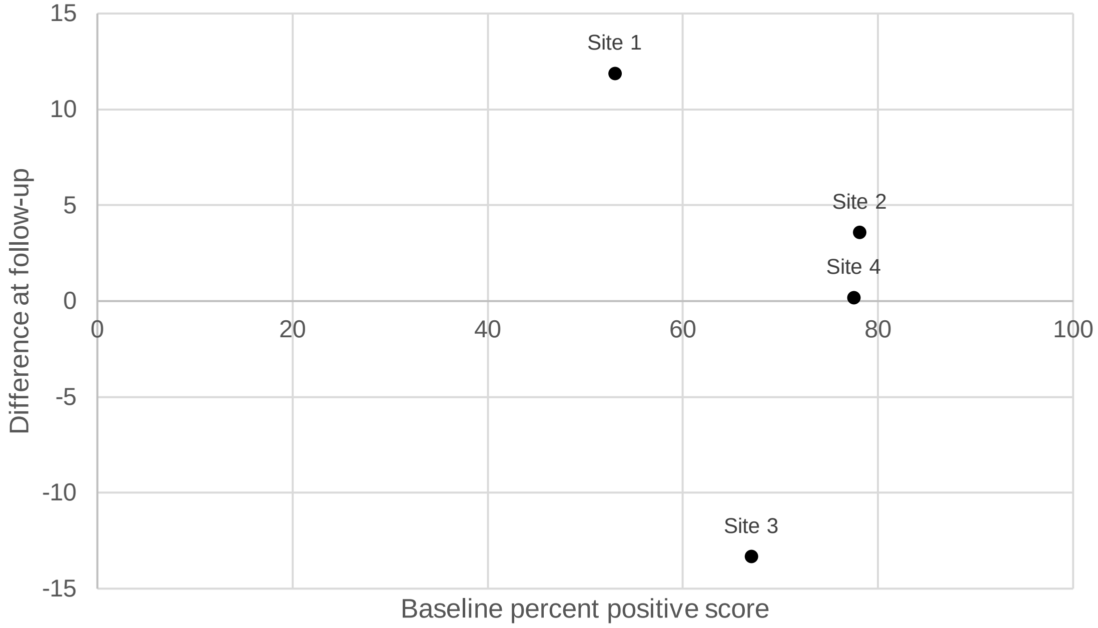

Supplemental Figure 1j. Staffing, percent positive score at baseline and difference at follow-up, by site

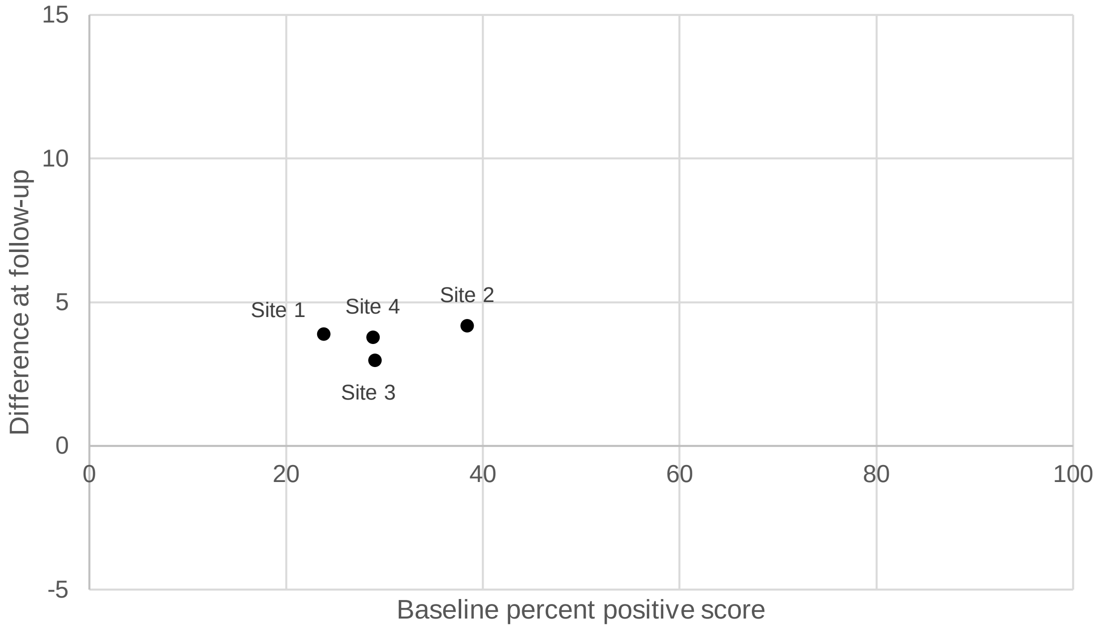

Supplemental Figure 1k. Handoffs and transitions, percent positive score at baseline and difference at follow-up, by site

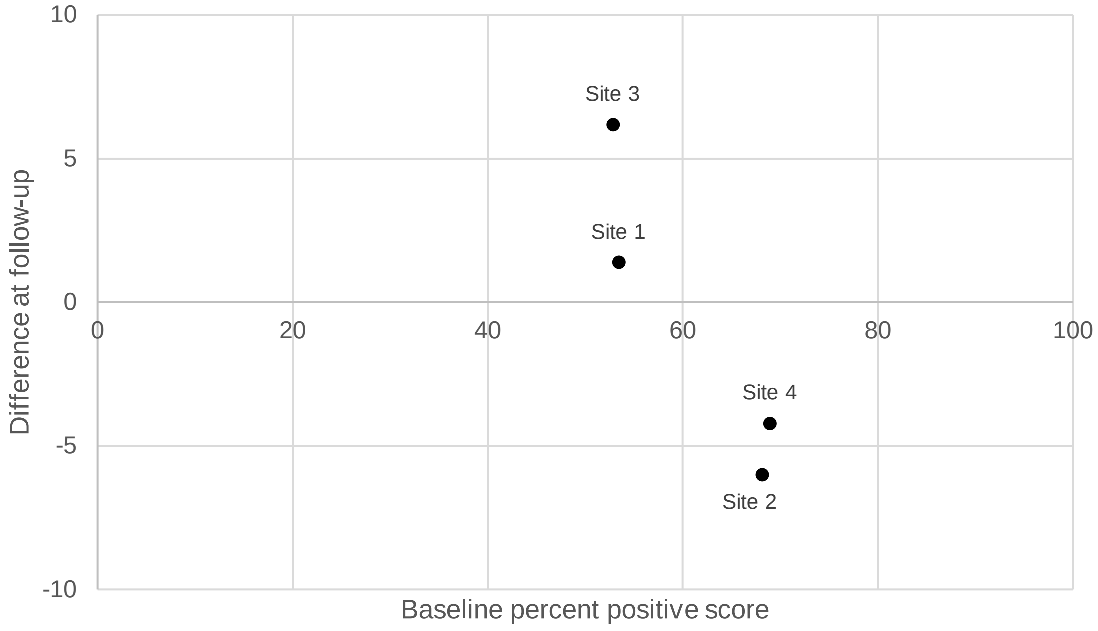

Supplemental Figure 1l. Nonpunitive response to errors, percent positive score at baseline and difference at follow-up, by site

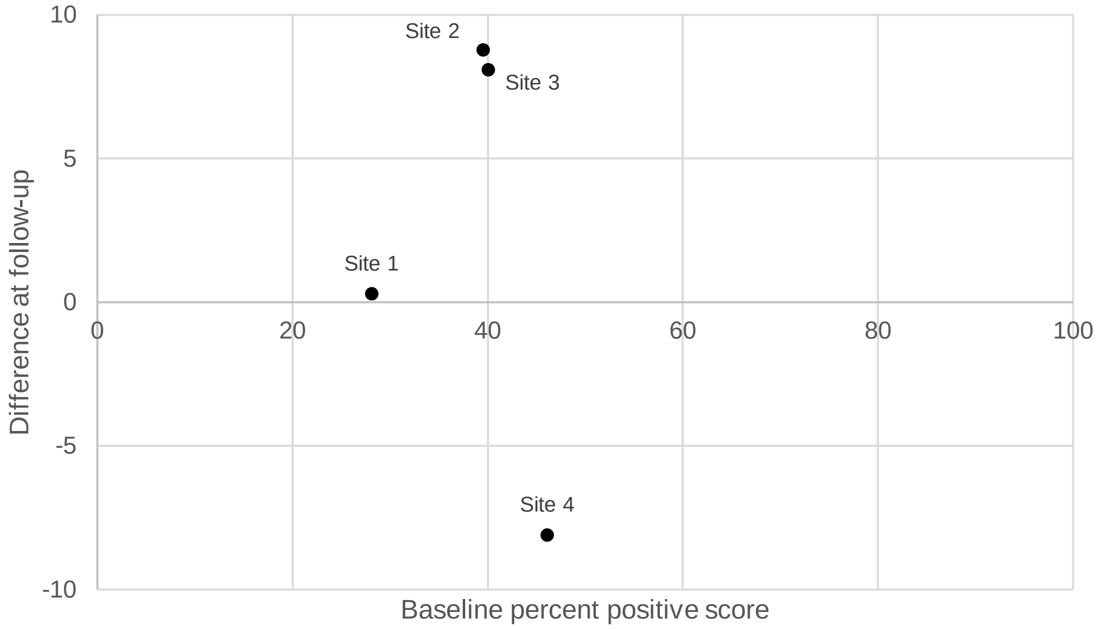

Supplemental Figure 1m. Patient safety grade, percent positive score at baseline and difference at follow-up, by site

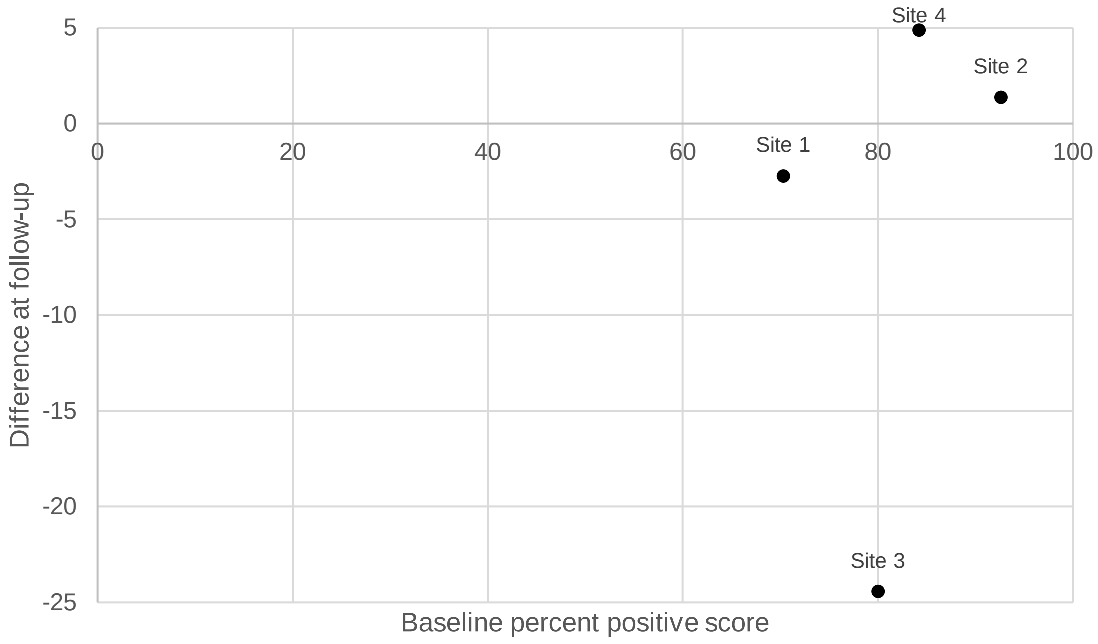

Supplement. JHU-SHEPheRD CUSP Tools

Tool 1. JHU-SHEPheRD Central Line Insertion Checklist

Tool 2. JHU-SHEPheRD Patient Safety Rounds

Tool 3. JHU-SHEPheRD Central Line Maintenance Audit Tool

Tool 4. JHU-SHEPheRD Injection Safety Assessment Tool

Name
Medical Record Number

NICU Central Arterial and Venous Catheter Insertion Checklist

1. **Today's Date: ____________ Time: _________**
2. **Person inserting line: __________________ Assistant: _______________ Observer:________________ Other: ____________________**
3. **Procedure is: □ Elective □ Emergent**
4. **Line Type: □ UAC □ UVC □ PICC [site:______________] □ Other _____**
5. **Number of attempts at this site: ____________**

Directions: Complete this form for all central line insertions; use a separate form for each site attempted.

Assistant (if not sterile) or Observer to tick the box when each task is performed. If the task is not performed, a comment must be added.

Line placement should be interrupted and corrected immediately if a deviation in infection control practice occurs.

	Critical Task	Yes	Yes with prompting	No	Comments
Prior to Insertion	'Time Out' performed immediately prior to procedure:	□	□	□	
	'Time Out' elements were verified by the inserter:				
	- patient ID	□	□	□	
	- site and side	□	□	□	
	- procedure	□	□	□	
	- all necessary equipment available	□	□	□	
	- patient position	□	□	□	
	- required barriers in place	□	□	□	
	- allergy or contraindication to iodine	□	□	□	
	- names/titles of participants	□	□	□	

	Critical Task	Yes	Yes with prompting	No	Comments
During Insertion	Hand hygiene is performed:	□	□	□	
	Infant is covered head-to-toe with a sterile drape:	□	□	□	
	All participating individuals at the sterile field are wearing proper attire:				
	- mask	□	□	□	
	- sterile gown	□	□	□	
	- hair cap with all hair covered	□	□	□	
	- sterile gloves	□	□	□	
	All individuals within 1 meter of procedure are wearing a mask:	□	□	□	
	Antiseptic used for skin preparation:	□ povidone-iodine (Betadine) solution<br>□ other			
	- Skin cleansed with spirit:	□	□	□	
	- Spirit allowed to air dry:	□	□	□	
	- Betadine painted on in circular motion out from, insertion site:	□	□	□	
	- Betadine solution air dry 2-min:	□	□	□	
	- No hand waving hands or blotting to speed drying antiseptic:	□	□	□	
	A sterile field was maintained throughout procedure:	□	□	□	

	Critical Task	Yes	Yes with prompting	No	Comments
After insertion	Type of sterile dressing applied to the site immediately after the procedure:	□ transparent dressing □ gauze<br>□ other, _______________<br>□ umbilical or not applicable			
	Caps and clamps in place on central-line:	□	□	□	
	Sharps and waste is disposed of safely:	□	□	□	
	Checklist completed by assistant/observer during the procedure:	□	□	□	
	Line position was confirmed:	□	□	□	
	Was the line repositioned?	□		□	Original ______ cm<br>Reposition ______cm

Place completed checklist in the Medical Record

JHU-SHEPheRD Patient Safety Rounds

<u>Guidance for planning organizing your rounds:</u>

1. **Set a *goal(s) for Patient Safety Rounds (choose those that fit):***

   □ Choose one patient to discuss in depth

   □ Round on several or all patients briefly

   □ Education session on safety topic

   □ Other: ______________________

2. ***Safety Rounds suggested steps:***

   - Notify the nurse manager, physician leaders and the Senior Executive prior to the scheduled rounds to:
     - Explain the goal and structure planned for the rounds
     - Describe the roles for each
     - Share the questions planned for rounds with the staff
     - Explain that rounds are a "safe place" for honest comments with the purpose of improving patient safety and there will be no repercussions on individuals
   - Upon arrival to the unit:
     - Contact the nurse manager and physician leader to help find one or two nurses and junior doctors in the area who can join
     - Nurse manager, physician leadership, and any available staff (e.g. pharmacist, respiratory therapists, technicians) are encouraged to participate
   - Begin the rounds (see sample agenda)
     - Explain that rounds are a "safe place" for honest comments with the purpose of improving patient safety and there will be no repercussions on individuals
     - The discussion is held on the floor in an open area to increase visibility
     - Specific questions about the factors or systems issues impacting safety are asked (in general or about specific patient(s)

3. ***Sample Agenda (30 min to 1 hour):***

   1. Gather participants
   2. Orient participants to goals and agenda for the rounds
   3. Review patient(s) (brief history, recent issues, current safety issues)
   4. Ask some of the suggested questions and seek input on issues and solutions from bedside staff (specific patient or general)
   5. Record findings and possible solutions on worksheet
   6. Discuss resources needed to solve the issue
   7. Review findings and assign follow-up tasks and dates
   8. Deliver training on specific safety issue (optional)

9. Senior Executive and/or leader of the rounds briefly describe a few of the important concepts that will lead to a safer environment (e.g. teamwork, open communication, and the importance of reporting and talking about near misses)

JHU-SHEPheRD Patient Safety Rounds Form

Date _____________

Attendees

Name	Role

□ Senior executive present □ Senior executive not present

Sample Questions

1. What aspects of this patient's care could be made safer?
2. Have you developed any personal practices that you do to specifically prevent making errors (memory aids, double-checking, forcing functions, etc.)?
3. Are there "work-arounds" or short-cuts you need to make when delivering care to this patient? Why?
4. In what ways are caregivers communicating with each other about this patient?
5. Are there any aspects of nurse-nurse /doctor-doctor / doctor-nurse communication that could be improved to make the care safer?
6. Have there been any instances where teamwork has been problematic with this patient?
7. Have there been any errors or "near misses" (that almost caused this patient harm but didn't)?
8. Do you feel you can "speak-up" when you identify a safety concern?
9. Has this patient's family voiced any safety concerns?
10. Has this patient's family voiced any safety concerns?
11. Is there anything that this group could do to prevent the next adverse event?
12. What do you think this unit could do on a regular basis to improve safety? For example, would it be feasible to discuss safety concerns, e.g., patients with same name, near misses that happened, etc., during report?

13. What specific intervention from leadership would make the work you do safer for patients?
14. What would make these executive Patient Safety Rounds more effective?

JHU-SHEPheRD Patient Safety Rounds Form

Findings:

Identified Issues	Potential/Recommended Solutions	Resources Needed	Follow-up: Responsible person(s)	Time frame

Discussion Points

□ Urgent issues identified

□ Follow-up items assigned

□ Achievements highlighted

□ Date/time set for next rounds

Feedback

□ Feedback to Department Head/NICU manager

□ The executive on rounds

□ Unit staff

□ CUSP meeting (highlight findings and ask for progress updates)

JHU-SHEPheRD Central Line Maintenance Audit Tool

NEED FOR LINE:
Was the need for this central line discussed today?
[ ] Yes [ ] No
[Useful only if the need for line discussion is documented or discussed at daily rounds. Should only be included if it is desired to work toward this implementing this key CL maintenance initiative.]

LINE DRESSING:
CLs and PICCs:
Is the CL or PICC dressing currently clean, dry and intact?
[ ] Yes [ ] No [ ] This is an umbilical line

Is the central line dressing dated with date of application?
[ ] Yes [ ] No [ ] This is an umbilical line

Was the dressing changed during this shift?
[ ] Yes [ ] No [ ] This is an umbilical line

If yes, dressing was changed because:

[ ] Dressing due to be changed (7 days for transparent dressing / 48 hours for gauze)
[ ] Dressing soiled, damp or not occlusive
[ ] Dressing overdue for a change

Umbilical lines: **Is the umbilical line clean, dry and not in nappy?**
[ ] Yes [ ] No [ ] N/A – this is <u>not</u> an umbilical line

ALL LINES:
Is the central line dated with date of insertion?
[ ] Yes [ ] No
[Useful only if lines are dated. Should only be included if it is desired to work toward this implementing this key CL maintenance initiative.]

TUBING:
Are all caps on all line access points that require them?
[ ] Yes [ ] No

Are all line connections properly fitting together and not wrapped?
[ ] Yes [ ] No

Is all central line tubing dated?
[ ] Yes [ ] No

Was central line tubing (main tubing and all additions) changed during this shift?
[ ] Yes
If yes, changed because:

[ ] Due to be changed
[ ] Overdue to be changed
[ ] No
If no, why?
[ ] Not due to be changed
[ ] Due to be changed but could not be completed

JHU-SHEPheRD Injection Safety Assessment Tool		
**Injection Practices and Sharps Safety**		
**Elements to be assessed**	**Assessment**	**Surveyor Notes**
**Injection Preparation**		
Injections are prepared in an area that has been cleaned with disinfectant and is free of contamination (e.g., visible blood, or body fluids).	o Yes<br>o No<br>o Unable to observe	
Injections are prepared using aseptic technique.	o Yes<br>o No<br>o Unable to observe	
Injections are prepared in an area that is separate to the immediate patient care area	o Yes<br>o No<br>o Unable to observe	
When drawing medications from vials, the top of the vial is swabbed with alcohol or alcohol-containing disinfectant before puncturing.	o Yes<br>o No<br>o Unable to observe	
The rubber septum on all medication vials, whether unopened or previously accessed, is disinfected with alcohol prior to piercing.	o Yes<br>o No<br>o Unable to observe	
Medication vials are entered with a new needle.<br><br>Note: Reuse of needles to enter a medication vial contaminates the contents of the vial, making the vial unsafe for use on additional patients. If the needle is reused to enter a vial to obtain additional medication for the same patient, the vial is discarded immediately.	o Yes<br>o No<br>o Unable to observe	
Medication vials are entered with a new syringe.<br><br>Note: Reuse of syringes to enter a medication vial contaminates the contents of the vial making the vial unsafe for use on additional patients. If a syringe is reused to enter a vial to obtain additional medication for the same patient, the vial is discarded immediately.	o Yes<br>o No<br>o Unable to observe	
Needles are used for only one patient.	o Yes<br>o No<br>o Unable to observe	
Syringes are used for only one patient (this includes manufactured prefilled syringes).	o Yes<br>o No<br>o Unable to observe	
**Single-dose vials**	o	
Medication vials labeled for single dose – single use are only used for one patient.	o Yes<br>o No<br>o Unable to observe	
If medication vials labeled for single dose are used for more than one patient, out of necessity, written protocol and visual instructions are in	o Yes<br>o No<br>o Unable to observe	

place to prevent cross contamination. These include:<br>- Vials only accessed in an area that is separate to the immediate patient care area<br>- Rubber septum wiped with alcohol before each access<br>- Access by multiple staff avoided<br>- New needle and new syringe used for every access<br>- Vial is clearly labelled with time of access<br>- Vial is not kept after it is accessed<br>- Use for single patient is preferred.		
Bags of IV solution are used for only one patient (and not as a source of flush solution for multiple patients).	o Yes<br>o No<br>o Unable to observe	
Medication administration tubing and connectors are used for only one patient.	o Yes<br>o No<br>o Unable to observe	
**Multi-dose vials**		
Only vials labeled as multi-dose are re-used.	o Yes<br>o No<br>o Unable to observe	
Multi-dose vials are stored correctly including in separate location to specimens and food.	o Yes<br>o No<br>o Unable to observe	
Multi-dose vials are dated when they are first opened and discarded within 28 days unless the manufacturer specifies a different (shorter or longer) beyond-use date for that opened vial.<br><br>Note: The beyond-use date is different from the expiration date printed on the vial by the manufacturer. The beyond-use date should never exceed the expiration date. The multi-dose vial can be dated by the hospital with either the date opened or the discard date as per hospital policy, as long as it is clear what the date represents and the same policy is used consistently.	o Yes<br>o No<br>o Unable to observe	
Multi-dose medication vials used for more than one patient are stored appropriately and do not enter the immediate patient treatment area (e.g., operating room, patient room, anesthesia carts).<br><br>Note: If multi-dose vials enter the immediate patient treatment area, they must be dedicated for single patient use and discarded immediately after use.	o Yes<br>o No<br>o Unable to observe	
Needles are not left in the septum of medication vials.	o Yes<br>o No<br>o Unable to observe	
Rubber septums of stored vials do not have visible holes in them.	o Yes<br>o No	

	o Unable to observe	
Ampules, once opened, are not kept and used for additional doses.	o Yes<br>o No<br>o Unable to observe	
**Sharps Disposal**		
All sharps are disposed of in puncture-resistant sharps containers.	o Yes<br>o No<br>o Unable to observe	
All sharps are disposed of at the point of use or carried to the sharps container in a puncture proof container.	o Yes<br>o No<br>o Unable to observe	
Sharps are never handled after use.	o Yes<br>o No<br>o Unable to observe	
Sharps containers are replaced when the fill line is reached.	o Yes<br>o No<br>o Unable to observe	
Sharps containers are disposed of appropriately as medical waste.	o Yes<br>o No<br>o Unable to observe	
Auto-disable needles are available.	o Yes<br>o No<br>o Unable to observe	
**Training, Competency, and Implementation of Policies and Procedures**		
Training is provided to all personnel who prepare and/or administer injections and parenteral infusions.	o Yes<br>o No	
Training is provided upon hire, prior to being allowed to prepare and/or administer injections and parenteral infusions.	o Yes<br>o No	
Training is provided at least annually.	o Yes<br>o No	
Training is provided when new equipment or protocols are introduced.	o Yes<br>o No	
Personnel are required to demonstrate competency with preparation and/or administration of injections and parenteral infusions following each training.	o Yes<br>o No	
Hospital maintains current documentation of competency with preparation and/or administration procedures for all personnel who prepare and/or administer injections and parenteral infusions.	o Yes<br>o No	
**Auditing Practices**		
Respondent can describe process used for audits of adherence to safe injection practices.	o Yes<br>o No	

Respondent can describe frequency of audits of adherence to safe injection practices.	o Yes<br>o No	
Respondent can describe process for improvement when non-adherence is observed.	o Yes<br>o No	
Respondent can describe how feedback from audits is provided.	o Yes<br>o No	
Respondent can describe frequency of feedback from audits.	o Yes<br>o No	

References:

CMS Survey: https://www.cms.gov/Medicare/Provider-Enrollment-and-Certification/SurveyCertificationGenInfo/Downloads/Survey-and-Cert-Letter-15-12-Attachment-1.pdf pp. 11-13

CDC ICAR: https://www.cdc.gov/infectioncontrol/pdf/icar/hospital.pdf Section F. Injection Safety

Strengthening Pharmaceutical Systems. Infection Control Assessment Tool, 2nd Edition. *Section 18 Injection Safety.* Submitted to the U.S. Agency for International Development by the Strengthening Pharmaceutical Systems Program. Arlington, VA: Management Sciences for Health; 2009

CDC standards: https://www.cdc.gov/infectioncontrol/guidelines/isolation/index.html
